# Transgenic force sensors and software to measure force transmission across the mammalian nuclear envelope *in vivo*

**DOI:** 10.1242/bio.059656

**Published:** 2022-11-09

**Authors:** Kelli D. Fenelon, Evan Thomas, Mohammad Samani, Min Zhu, Hirotaka Tao, Yu Sun, Helen McNeill, Sevan Hopyan

**Affiliations:** ^1^ Program in Developmental and Stem Cell Biology, Research Institute, The Hospital for Sick Children, Toronto, ON M5G 0A4, Canada; ^2^ Department of Molecular Genetics, University of Toronto, Toronto, ON M5S 1A8, Canada; ^3^ Department of Mechanical and Industrial Engineering, University of Toronto, Toronto, ON M5S 3G8, Canada; ^4^ Department of Developmental Biology, Washington University, St. Louis, MO 63110, USA; ^5^ Lunenfeld-Tanenbaum Research Institute, Toronto, ON M5G 1X5, Canada; ^6^ Division of Orthopaedic Surgery, Hospital for Sick Children and University of Toronto, ON M5G 1X8, Canada

**Keywords:** FLIM software, FRET-based force sensors, Nemp1, Mouse embryo, Nuclear envelope, Transgenic, Nesprin-2G, Limb bud, Nuclear mechanotransduction

## Abstract

Nuclear mechanotransduction is a growing field with exciting implications for the regulation of gene expression and cellular function. Mechanical signals may be transduced to the nuclear interior biochemically or physically through connections between the cell surface and chromatin. To define mechanical stresses upon the nucleus in physiological settings, we generated transgenic mouse strains that harbour FRET-based tension sensors or control constructs in the outer and inner aspects of the nuclear envelope. We knocked-in a published esprin-2G sensor to measure tensions across the LINC complex and generated a new sensor that links the inner nuclear membrane to chromatin. To mitigate challenges inherent to fluorescence lifetime analysis *in vivo*, we developed software (FLIMvivo) that markedly improves the fitting of fluorescence decay curves. In the mouse embryo, the sensors responded to cytoskeletal relaxation and stretch applied by micro-aspiration. They reported organ-specific differences and a spatiotemporal tension gradient along the proximodistal axis of the limb bud, raising the possibility that mechanical mechanisms coregulate pattern formation. These mouse strains and software are potentially valuable tools for testing and refining mechanotransduction hypotheses *in vivo*.

## Teaser

**Summary:** We introduce transgenic and software tools to measure tensions across the outer and inner nuclear envelope in mice. These tools facilitate our ability to test mechanotransduction hypotheses *in vivo*.

## INTRODUCTION

The cell nucleus experiences physical forces in a manner that has the potential to alter chromatin conformation (Irianto et al., 2017; Iyer et al., 2012), gene expression (Fenelon and Hopyan, 2017; Jain et al., 2013; Le et al., 2016; Tajik et al., 2016) and downstream consequences such as cell identity (Shin et al., 2013; Swift et al., 2013) and movement (Harada et al., 2014; Luxton et al., 2010; Petrie et al., 2014). Transduction of force is an alternative and more rapid means of transmitting signals to the nucleus than the diffusion of biochemical signals (Na et al., 2008). By advancing mechanisms of mechanical signal transduction, we can potentially generate and test a broad range of biological hypotheses including those concerning development and disease (Aureille et al., 2017; Cho et al., 2017; Graham and Burridge, 2016; Maurer and Lammerding, 2019; Uhler and Shivashankar, 2017). For example, forces that shape embryonic tissues might co-regulate cell differentiation (Maurer and Lammerding, 2019) and/or the expression of patterning genes (Papageorgiou, 2011), and feedback from pathologically stiff tissue environments might alter gene expression to promote the progression of chronic inflammation and neoplasia (Maurer and Lammerding, 2019).

Many of the key methods required for this area of research, such as quantitative analysis of RNA and protein expression at single cell resolution (Lamanna et al., 2020), determination of chromatin conformation (Gentile et al., 2019; Mateo et al., 2019), and measurement of nuclear (Davidson et al., 2019; Liu et al., 2014; Wang et al., 2019, 2018a) and tissue (Serwane et al., 2017; Wang et al., 2018b; Zhu et al., 2020) viscoelastic properties, have advanced considerably. In contrast, tools to measure forces experienced by nuclei are only just emerging. Recently, genetically encoded sensors to measure force transduction across the nuclear periphery have been described for *in vitro* studies (Arsenovic et al., 2016; Carley et al., 2021). Model organisms expressing force sensors would be useful to define the extent to which actomyosin or external stresses that deform the nucleus (Booth-Gauthier et al., 2012; Cho et al., 2019; Stewart et al., 2019) are transmitted across the nuclear envelope in developmental and other physiological contexts. Recent attempts to utilise transgenic tension sensors *in vivo* have generated both promising (Tao et al., 2019) and discouraging (Eder et al., 2017) outcomes. Challenges inherent to employing Förster resonance energy transfer (FRET)-based sensors *in vivo* need to be addressed before they can be applied robustly in animals.

Mechanical connectivity between the nucleus and cytoskeleton with extracellular matrix or neighbouring cells is increasingly understood. Underlying that connectivity are physical attachments of the cytoskeleton to the nuclear envelope through the linker of nucleoskeleton and cytoskeleton (LINC) complex that links f-actin, microtubules, and intermediate filaments to the nuclear lamina (Lombardi and Lammerding, 2011). Mammalian esprin (nuclear envelope with spectrin repeats) proteins that reside across the outer nuclear membrane (ONM) bind cytoskeletal filaments such as f-actin and are anchored to the inner nuclear membrane (INM) by SUN1/2 (Sad1, UNsC84) proteins (Carley et al., 2021; Lombardi et al., 2011; Starr, 2011). The nuclear lamina lines the interior aspect of the INM. Lamin protein filaments polymerise to form a mesh that largely determines the stiffness of the nucleus, in part by tethering chromatin (Harada et al., 2014; Schreiner et al., 2015; Vergnes et al., 2004; Zheng et al., 2018). Chromatin binding is mediated, in part, by embedded proteins that harbour an LEM (LAP2-emerin-MAN1) domain (Barton et al., 2015). emp1 is a five-pass INM protein with a C-terminal (nucleoplasmic) putative BAF-binding domain (Mamada et al., 2009; Shibano et al., 2015) that binds double stranded DNA in a sequence-independent manner (Mamada et al., 2009) and interacts with emerin and other chromatin-binding components of the nuclear lamina (Tsatskis et al., 2020). In response to tissue stiffness and forces exerted upon the nucleus, lamin-A, emerin and NEMP1 modulate nuclear physical properties (Guilluy et al., 2014; Langevin et al., 2010; Swift and Discher, 2014; Tsatskis et al., 2020), shape (Jain et al., 2013; Langevin et al., 2010), chromatin organisation (Iyer et al., 2012; Le et al., 2016), and transcription (Jain et al., 2013; Le et al., 2016; Tajik et al., 2016). Through these nuclear envelope complexes, forces may directly alter chromatin conformation. To distinguish direct effects from indirect force-induced signalling cascades, it would be helpful to measure forces transmitted across the LINC complex and the INM/lamina.

Here we present a pair of knock-in, FRET-based sensors that permit the measurement of tensions across the LINC complex component esprin-2 giant (esprin-2G) and the INM protein NEMP1 in mice. The former was previously generated for use *in vitro* (Arsenovic et al., 2016), whereas the latter is an entirely new sensor. Control transgenic strains were generated from FRET donor-only and headless/tailless constructs, and the expressed sensor proteins localise appropriately. We also introduce software that mitigates challenges imposed by autofluorescence and low signal-to-noise ratio to facilitate the fitting of fluorescence decay curves generated by fluorescence lifetime imaging microscopy (FLIM) of living embryos. The sensors respond to cytoskeletal relaxation and externally applied stretch by exhibiting increased fluorescence lifetime/tension means or ranges. By employing the mouse embryo for proof-of-principle analysis, we identify spatial and temporal differences in tensions across the nuclear envelope that correspond to nuclear roundness and tissue stiffness.

## RESULTS

### Generation of tension sensor mice

**Fig. 1.**
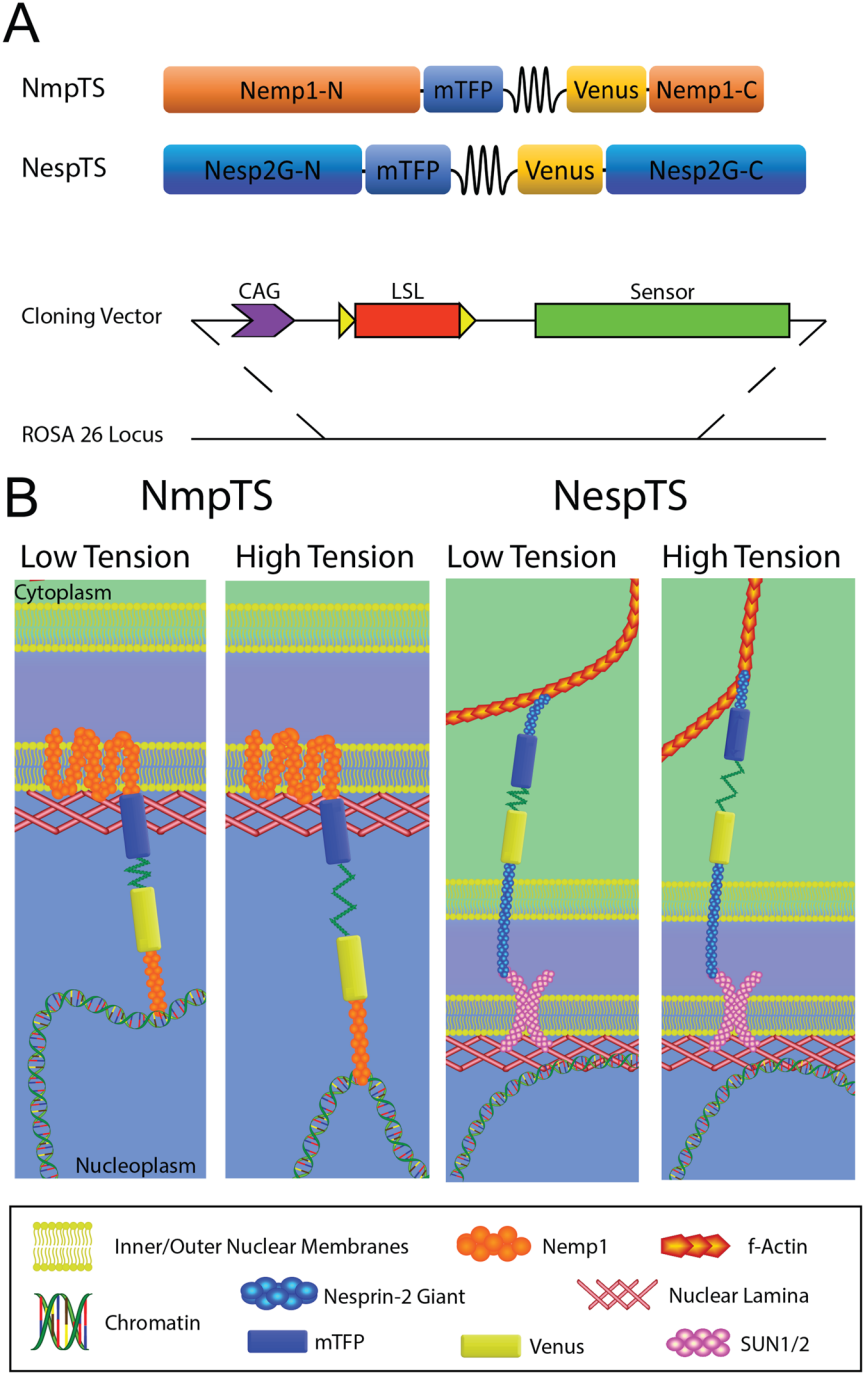
**Nuclear envelope tension sensors.** (A) The tension sensors consist of a FRET pair, mTFP and Venus, separated by flagelliform peptide springs inserted between the transmembrane and BAF binding domains of Nemp1, and between the SUN and actin binding domains of Nesprin-2G. To ensure robust transcription and enable conditional expression, a CAG promoter and lox-STOP-lox cassette were inserted upstream of the sensor sequences, respectively. The sensors were knocked into the *ROSA26* locus of the mouse genome via homologous recombination in ES cells. (B) Schematic of sensor function. NmpTS should report tension between the inner nuclear membrane and chromatin, whereas NespTS should report tension through the LINC complex.

We wished to generate knock-in mouse strains that harbour tension sensors within the outer and inner aspects of the nuclear envelope. To measure force transmission across the LINC complex, we generated a mouse strain capable of conditionally expressing a previously published tension sensor (NespTS) (Arsenovic et al., 2016). The construct consists of a FRET-based tension sensing module (Grashoff et al., 2010) between the actin- and SUN-binding domains of esprin-2G (Fig. 1A,B). A previously described headless control construct that lacks the actin binding domain of esprin-2G (NespHL) was prepared as a separate transgenic construct (Fig. S1A).

**Fig. 2.**
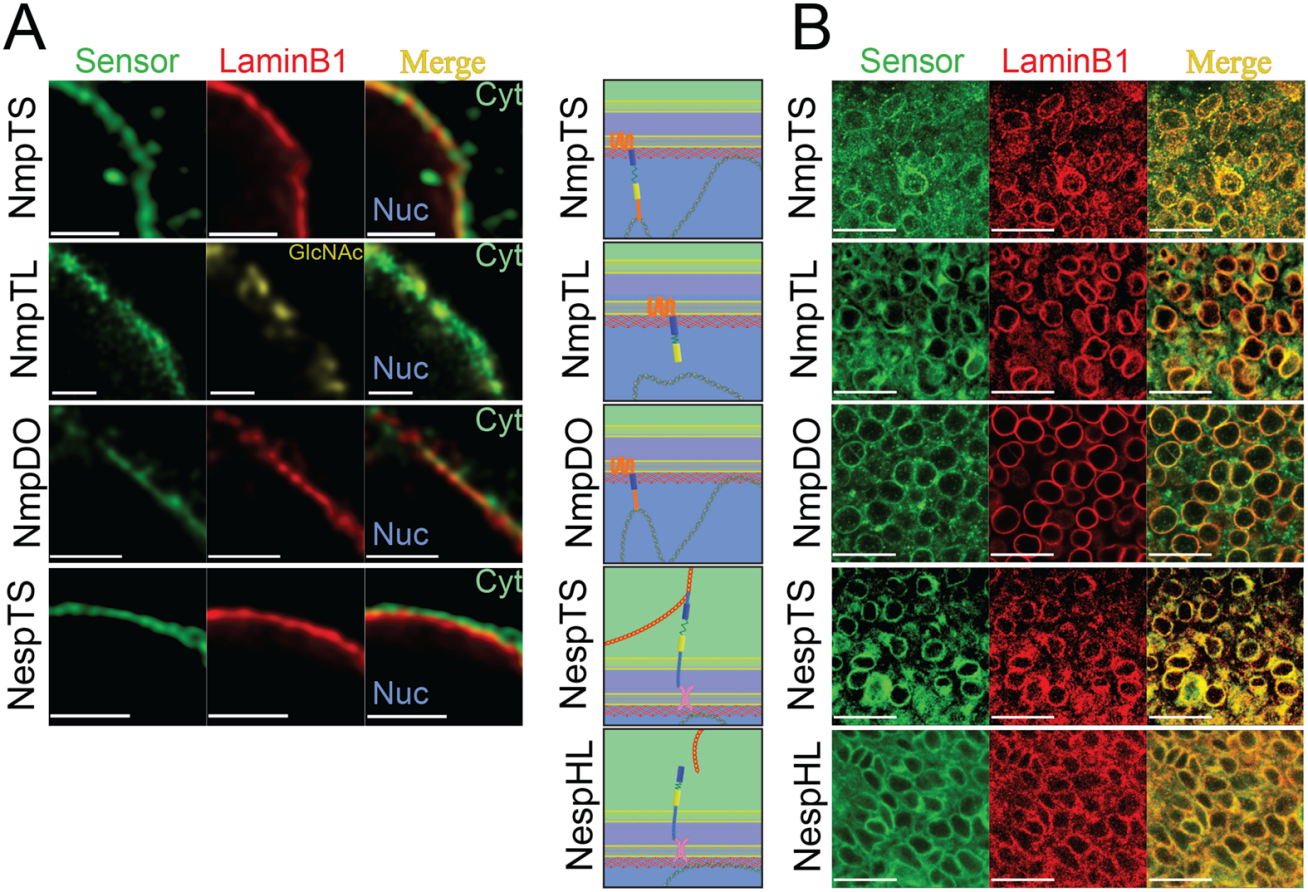
**Tension sensors localise to appropriate nuclear envelope locations.** (A) Super resolution STED microscopy demonstrates INM localisation of Nemp1 sensor constructs in fixed 293T cells. Nemp1 constructs colocalised with INM protein aminB1 (red) and nuclear pore marker O-linked N-acetylglucosamine (yellow), while NespTS localised external to aminB1. Scale bars: 2 µm. (B) Schematics illustrate the anticipated location and function of each construct. Confocal images of fixed E9.5 embryo forelimb mesenchyme demonstrate nuclear envelope localisation of the sensor constructs *in vivo* by immunofluorescence. Scale bars: 25 µm.

Since chromatin is a structural component of the nucleus and chromatin architecture regulates transcription, we considered INM tension sensor candidates that link the membrane to chromatin and chose the INM protein. A previously characterised FRET-based tension sensor module (Grashoff et al., 2010) was inserted between the final trans-membrane domain and the C-terminal putative BAF-binding domain (NmpTS - Fig. 1A,B; Fig. S1B). Control constructs, FRET donor-only (NmpDO) and tailless (NmpTL), were also prepared (Fig. S1A). The tension sensor and control constructs were transiently expressed *in vitro*. Super resolution stimulated emission-depletion (STED) microscopy revealed appropriate co-localisation of NmpTS and controls with laminB1 (reflecting INM location), and that of NespTS external to laminB1 (reflecting ONM location, Fig. 2A).

Previous findings suggested that *in vivo* imaging of FRET-based tension sensors is impaired by low signal-to-noise ratio (Eder et al., 2017). For this reason and to avoid developmental stage and tissue expression disparities, we employed a CAG promoter ensure robust expression of the five sensor or control reporters. A lox-stop-lox sequence interrupted the promoter-gene junction to allow for Cre-based conditional expression (Fig. 1A). Constructs were knocked-into the ROSA26 locus by homologous recombination in ES cells and chimeric mice were generated by ES cell-morula aggregation. Following germ-line transmission, mice were bred to pCX-NLS-Cre or CMV-Cre mice to generate ubiquitously active strains (henceforth referred to by their construct name).

By confocal fluorescence microscopy, we confirmed that all five sensor and control proteins localised to the nuclear envelope *in vivo* (Fig. 2B). FLIM signal was readily detectable within different tissues and under experimental perturbations *in vivo* (Fig. S2A). Importantly, NmpTS and NespTS caused no measurable alteration of nuclear shapes compared to H2B-GFP and comparisons of nuclei marked with either NmpTS or NespTS revealed a small difference in nuclear size that is expected based on their relative positions at the INM or ONM in E9.5 distal FL mesenchyme (Fig. S2B-D).

Adult mice from all five new strains that ubiquitously expressed the transgenes were morphologically normal, fertile and had normal body weight (Fig. S3A,B). Nesprin-2 null mice are known to be viable and fertile (Zhang et al., 2007). The NespTS can functionally rescue centrosomal orientation and nuclear movement defects of NIH3T3 fibroblasts depleted of endogenous esprin-2 *in vitro* (Arsenovic et al., 2016). To test the functional equivalency of NmpTS to NEMP1, we bred the sensor and control strains onto a *Nemp1* null background (Tsatskis et al., 2020). Presence of the NmpDO, but not NmpTS, transgene rescued the otherwise markedly diminished litter size of females at E8.5-9.5 (Fig. S3C,D). These findings imply the length or nature of the tension sensor domain, rather than the level of expression or disruption of the endogenous amino acid sequence, most affects function of the protein. We concluded that NempTS is not functionally equivalent to NEMP1 but could be a useful measurement tool.

### An *in vivo* analysis toolkit

**Fig. 3.**
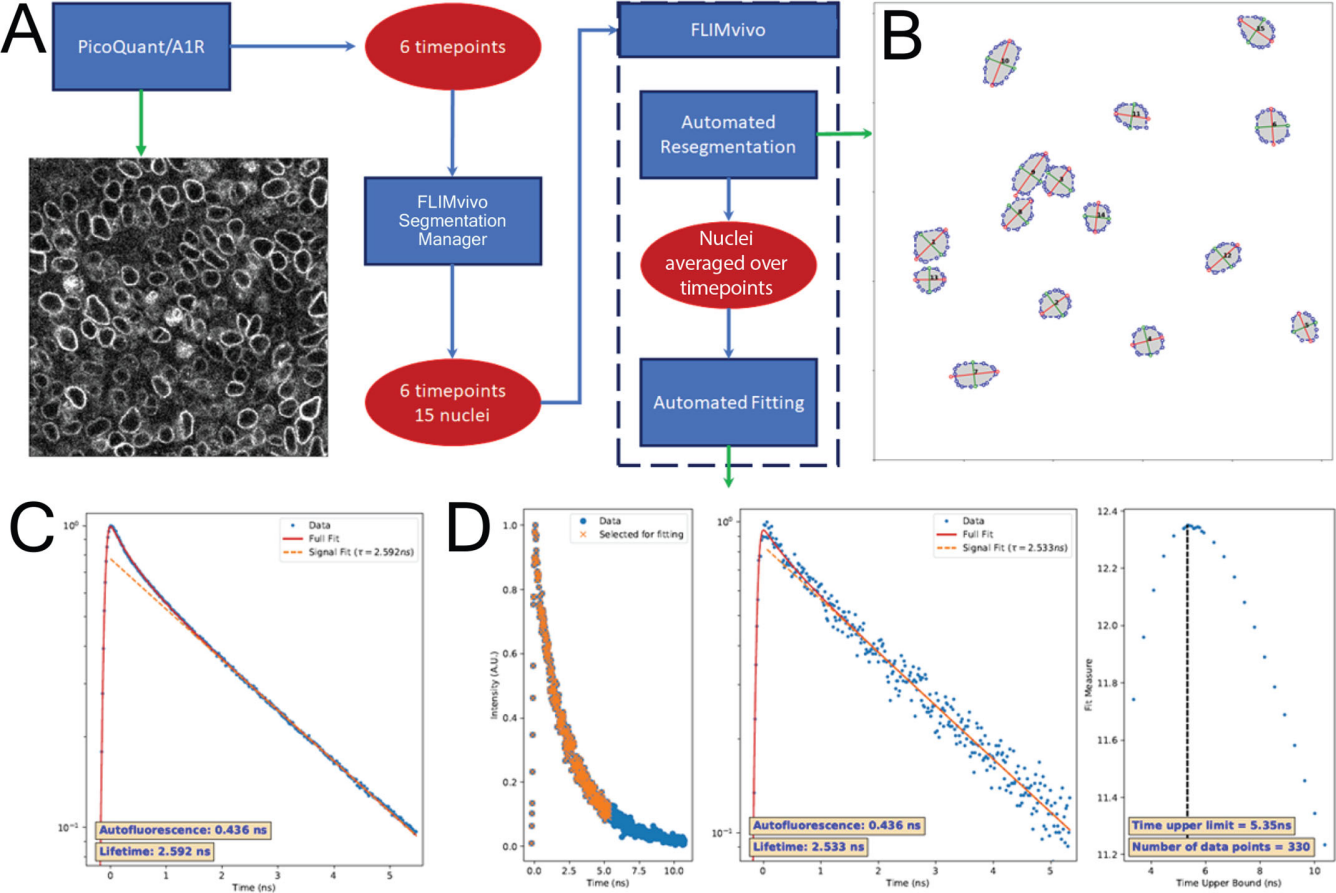
**FLIMvivo fluorescence lifetime fitting pipeline.** (A) To average forces over time and remove tissue level extremums, acquired FLIM images were saved in PicoQuant acquisition software at six timepoints (every 2 min, 10 min total). These images were semi-automatically segmented for regions of interest (we chose a target of 15 nuclear membranes) using our segmentation manager. (B) FLIMvivo re-segmented nuclei and assessed the aspect ratio (roundness) for each one. The software then combined FLIM data for each nucleus across all timepoints for fitting. (C) Sample NmpTS data illustrate how FLIMvivo used all data from FLIM images per dataset to fit for instrument response and autofluorescence (red fit line), and determined whether to use a mono- or bi-exponential fit for each dataset (orange dotted line). (D) Sample NmpTS data illustrate how each segment was fit individually and lifetime measurements were generated. Left panel: FLIMvivo output showing the data used for lifetime fit. Middle panel: FLIMvivo output showing the lifetime fit curve (red line), donor fit (orange dotted line), and lifetime values (bottom left). Right panel: FLIMvivo output plotting the goodness-of-fit of sequential fits used to threshold background light.

Acquiring force data in live mouse embryos is conceptually ideal because it reflects a physiologically relevant environment, but analysis by FLIM or FRET is challenging due to low signal-to-noise ratio *in vivo*. Auto-fluorescence, light scatter, and low photon counts are obstacles to reliable data analysis. To mitigate these challenges, we developed software to semi-automatically segment regions of interest such as nuclei and optimally fit fluorescence decay data acquired *in vivo* (FLIMvivo) (Fig. 3A-D; Fig. S4A,B). We chose to analyse sensor data using FLIM because the method is independent of fluorescence intensity unlike ratiometric FRET analysis. FLIM data were acquired at 2-min intervals under live conditions (Wyngaarden et al., 2010). To maximise signal-to-noise, nuclear envelopes were isolated from the resultant images using a segmentation manager we prepared for FLIMvivo (Fig. S5A). Difference in the segmentation efficiency between the two tension sensor strains was not significant (Fig. S5B). The program compiled data across six time points to increase photon counts and to improve fit quality for individual nuclei (Fig. 3C,D; Fig. S4B).

There are two principal sources of noise in our setting. A quick fluorescence decay that is likely due to autofluorescence can artificially decrease the measured fluorescent lifetime, and background, or scattered, light can artificially increase the fit value for measured lifetime unless addressed appropriately (Fig. S4C). In addition, signal-to-noise ratios between samples and tissues cannot be assumed to be consistent. Managing autofluorescence is complicated by the fact the fast mode directly following the laser pulse is often not much longer than the timescale of the instrument response function. There is not an obvious way to fit individual curves to account for these problems using a simple tail fit because it is not clear where each tail should be cut. Furthermore, any given segment generally does not have enough data to simultaneously fit for autofluorescence and FRET signal time constants as they are poorly correlated. We overcame these challenges by recognising that autofluorescence is likely unrelated to our sensors, thereby allowing us to fit for the autofluorescence signal using the entire field of view and by using a full convolution fit that properly defines the instrument response (Fig. S4C). Once these features were accounted for, individually segmented regions of interest could be analysed (Fig. 3C,D).

### Testing sensor responses *in vivo*

**Fig. 4.**
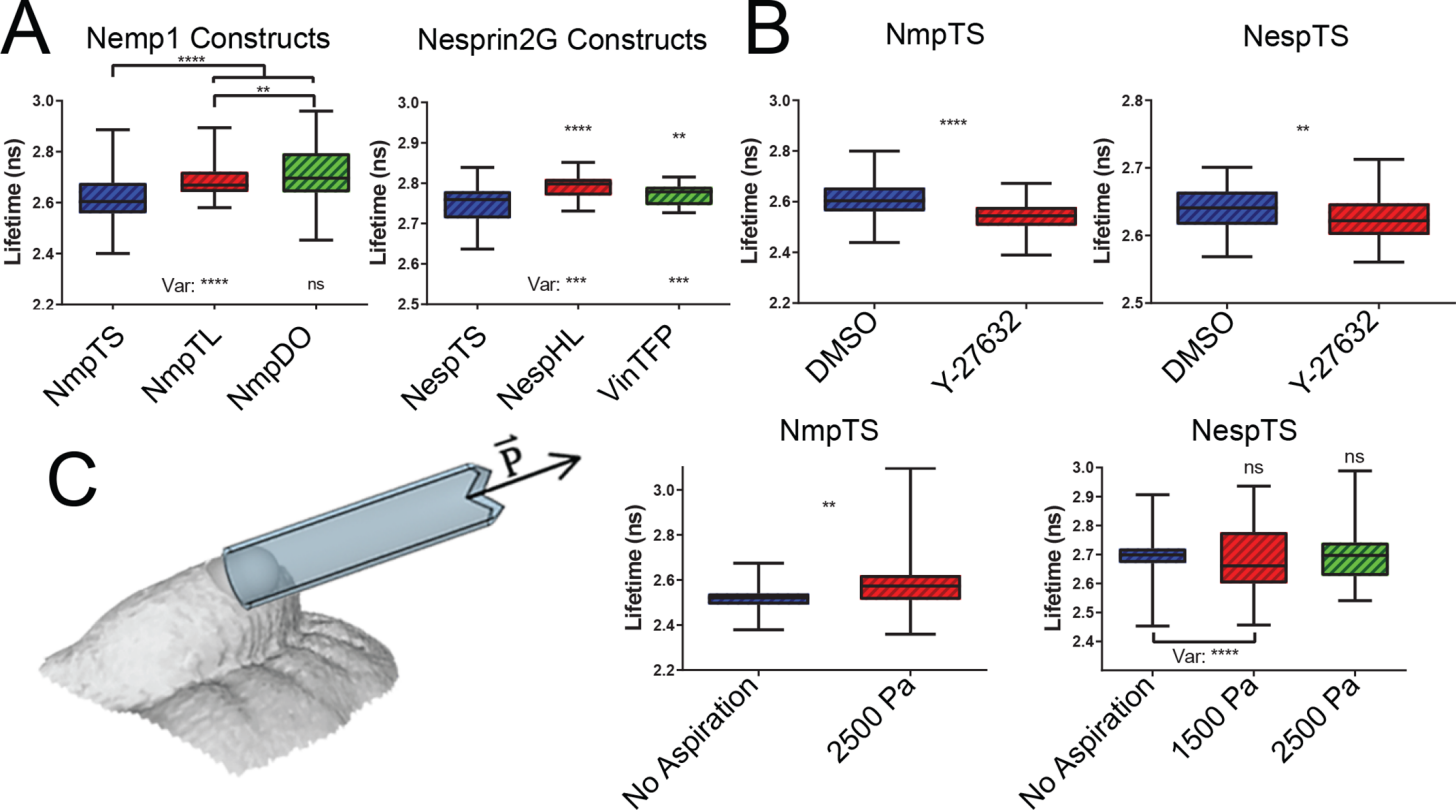
**Nuclear envelope tension sensor responses to cytoskeletal and external stresses *in vivo.*** (A) *In vivo* FLIM measurements demonstrated lower NmpTS and NespTS lifetimes than nucleoplasmic (NmpDO) or cytoplasmic (VinTFP) donor only controls [*t*-test (as for all comparisons unless otherwise stated): NmpTS: *P*<0.0001; NespTS: *P*=0.0042]. NmpTL lifetimes were significantly lower than those of NmpDO (*P*=.0084). NmpTL and NespHL report significantly less variance than NmpTS and NespTS, respectively [*F* test (as for all variances): NmpTL: *P*<0.0001; NespHL: *P*=0.0008; NmpDO: *P*=0.3964; VinTFP: *P*<0.0001]. NmpTS: *n*=68 nuclei from five embryos; NmpTL: *n*=110 nuclei from eight embryos; NmpDO: *n*=126 nuclei from nine embryos; NespTS: *n*=105 nuclei from seven embryos; NespHL: *n*=45 nuclei from three embryos. (B) Treatment of embryos in roller culture with Y-27632 significantly decreased tension across both NmpTS and NespTS (NmpTS: *P*<0.0001; NespTS: *P*=0.0066). NmpTS: DMSO (carrier): *n*=105 nuclei from seven embryos; 5 µM (Y-27632): *n*=45 nuclei from three embryos; NespTS: DMSO: *n*=45 nuclei from three embryos: *n*= 60 nuclei from four embryos (C) Precise micro-aspiration of live E9.5 embryo distal forelimb buds using a 90 µm diameter capillary tube at 2500 Pa significantly increased mean lifetime/tension across NmpTS (*P*=0.0098), but not across NespTS at 2500 Pa (*P*=0.9696) or 1500 Pa (*P*=0.7920). Aspiration increased the variance of lifetimes reported by NmpTS and NespTS (*F* test, *P*<0.0001, *P*<0.0001). NmpTS Pre (no)-aspiration: *n*=65 nuclei from five embryos; NmpTS 2500 Pa: *n*=43 nuclei from five embryos; NespTS Pre-aspiration: *n*=118 nuclei from eight embryos; NespTS 1500 Pa: *n*=43 nuclei from four embryos; NespTS 2500 Pa: *n*=41 nuclei from four embryos.

*** ***We tested individual sensor responses *in vivo* using E9.5 wild-type (*Nemp1^+/+^*) distal limb bud mesoderm as a model system due to its accessibility and well-studied patterning characteristics in intact embryos under live conditions. Relative to NmpTS, NmpDO exhibited higher mean lifetime values, as expected. NmpTL and NespHL reported low lifetime values relative to NmpDO and previously published donor only mTFP measurements (Grashoff et al., 2010; Tao et al., 2019) with low standard deviation, as expected. Appropriately, NmpTS and NespTS reported a wider range of lifetimes than those of NmpTL and NespHL but, unexpectedly, reported lower means compared to their tailless and headless controls (Fig. 4A). The relatively narrow and consistent lifetime ranges reported by the tailless and headless controls between tissues (Fig. S6A-C) suggest that, unlike their full-length counterparts (shown in different tissues below), these controls do not respond to tension but are trapped in a semi-stretched state. It was recently shown that the position of the sensor module within a similar esprin-2G construct used *in vitro* can unexpectedly decrease FRET index but does not compromise the full-length sensor readout (Déjardin et al., 2020). For future applications, we will preferentially compare full length tension sensor readings to donor-only control means, whereas the tailless and headless controls will be useful for comparisons of lifetime range.

We next sought to alter nuclear characteristics *in vivo*. To test the effect of actomyosin inhibition upon our transgenic sensors, we treated embryos in roller culture with Rho-associated kinase inhibitor Y-27632. Compared to carrier-treated controls, Y-compound treatment reduced donor lifetime across both sensors (Fig. 4B). Inhibition of actin polymerisation by treatment with Cytocalasin-D produced similar results across NmpTS (Fig. S6D). It is recognised that cytoskeletal forces are exerted through the LINC complex and this finding confirms those forces are transmitted across the inner nuclear membrane. Pharmacological perturbations of global chromatin architecture by treatment with either doxorubicin (Waldes and Center, 1981) or 2-deoxyglucose and sodium azide (Lléres et al., 2009; Visvanathan et al., 2013) significantly reduced tension across NmpTS (Fig. S6E,F). Those data suggest that condensation and fragmentation of chromatin globally relaxes the tension module.

To determine if our sensors are responsive to exogenous tensile stress, we applied precisely controlled micro-aspiration of the E9.5 distal forelimb bud as we have done previously (Wen et al., 2017). Aspiration up to 2500 Pa, but not treatment with Y-27632, elongated cells (Fig. 4C; Fig. S2A, S7A-E). When aspiration was maintained during FLIM acquisition, mean lifetime/tension across NmpTS was increased (Fig. 4C). For NespTS, aspiration widened the distribution of lifetime values but did not change the mean (Fig. 4C). Possible explanations for this finding are that LINC complex tension is a function of perinuclear orientation with regard to the axis of stress or that the complex remodels to dissipate stress. The two sensors, therefore, report related but distinct aspects of force transmission across the nuclear envelope.

### Measuring nuclear forces in developing tissues

**Fig. 5.**
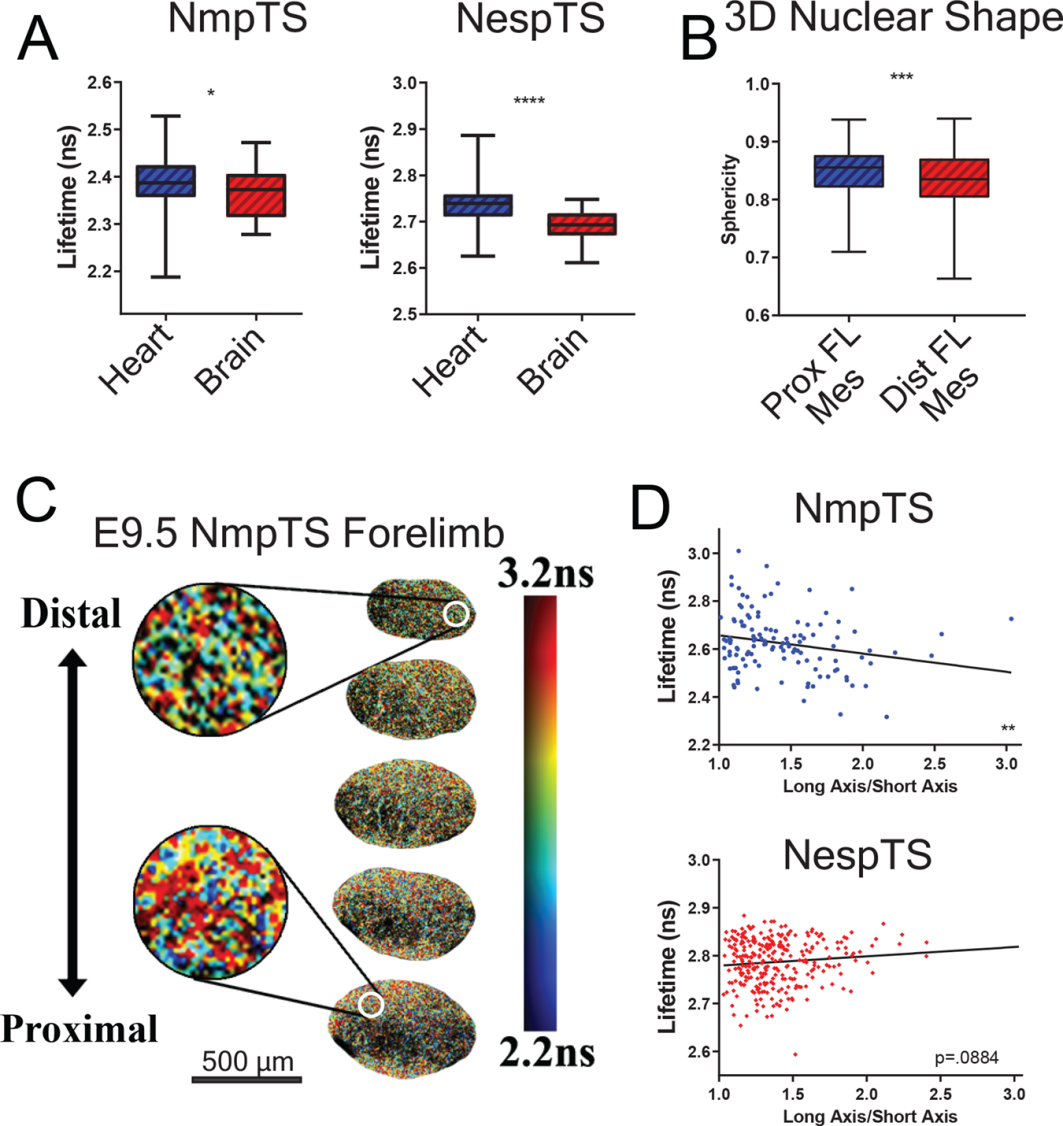
**Spatial variation of nuclear envelope tensions in the mouse embryo.** (A) Lifetime/force across NmpTS and NespTS is significantly higher in the beating myocardium (Heart) than in the anterior forebrain (Brain) (NmpTS: *P*=0.0435; NespTS: *P*<0.0001). NmpTS: Brain: *n*=32 nuclei from three embryos; Heart: *n*=44 nuclei from three embryos; NespTS: Brain: *n*=45 nuclei from three embryos; Heart: *n*=40 nuclei from three embryos. (B) At E9.5, nuclei in the proximal region are significantly more spherical than those in the distal region (*P*=0.0008, Prox FL Mes: *n*=239 nuclei from three embryos; Dist FL Mes: *n*=327 nuclei three embryos; Mann–Whitney test). (C) Colour-coded representation of NmpTS lifetimes/forces along the proximodistal axis of E9.5 limb bud mesoderm (generated from confocal FLIM images processed in FLIMfit). (D) Nuclear roundness correlates with increased lifetime/force across NmpTS (slope: −0.07638; *F* test: *P*=0.0106) but not NespTS (slope: 0.01940; *F* test: *P*=0.0884).

One aim of generating knock-in versions of nuclear tension sensors is to link spatiotemporal changes in morphogenetic forces to gene expression. As a first step toward that goal, we tested the output of our sensors in different tissues and at different times. The heart and brain are potentially useful proof-of-principle candidates because they are structurally different tissues that exhibit distinct mechanical properties (Raghunathan et al., 2017; Swift and Discher, 2014). Both NempTS and NespTS embryos reported higher mean forces within the beating E9.5 myocardium than from the E9.5 anterior forebrain (Fig. 5A), an observation that is consistent with the contractile behaviour of cardiomyocytes.

**Fig. 6.**
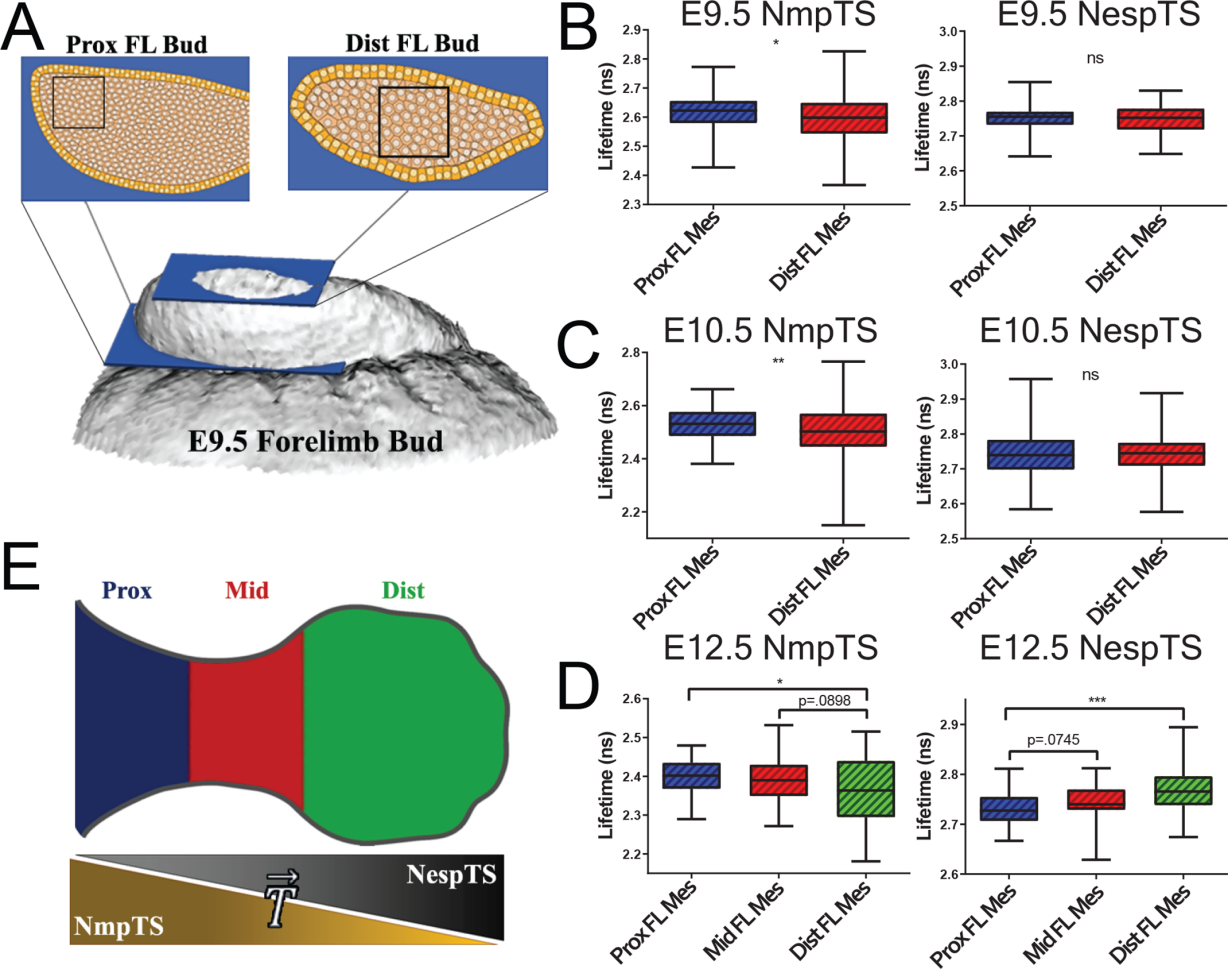
**Spatio-temporal variation of nuclear envelope tensions in the limb bud.** (A) Schematic representation of the proximal and distal regions of the early limb bud examined by FLIM. (B,C) Forces exerted across NmpTS, but not NespTS, are significantly higher in the proximal than the distal region of the forelimb bud at E9.5 and E10.5. For NmpTS: at E9.5: *P*=0.0265, Prox FL Mes: *n*=68 nuclei from five embryos, Dist FL Mes: *n*=84 nuclei from five embryos; at E10.5: *P*=0.0015: Prox FL Mes: *n*=89 nuclei from six embryos, Dist FL Mes: *n*=179 nuclei from six embryos. For NespTS: at E9.5: *P*=0.4278, Prox FL Mes: *n*=105 nuclei from seven embryos, Dist FL Mes: *n*=105 nuclei from seven embryos; E10.5: *P*=0.8120, Prox FL Mes: *n*=55 nuclei from three embryos, Dist FL Mes: *n*=55 nuclei from three embryos. (D) At E12.5, forces exerted across NmpTS decrease in the distal forelimb, whereas those exerted across NespTS increase distally. For NmpTS: Prox versus Dist FL Mes: *P*=0.0157; Prox FL Mes: *n*=45 nuclei from three embryos, Mid FL Mes: *n*=45 nuclei from three embryos, Dist FL Mes: *n*=45 nuclei from three embryos. For NespTS: Prox versus Dist FL Mes: *P*=0.0002; Prox FL Mes: *n*=45 nuclei from three embryos, Mid FL Mes: *n*=45 nuclei from three embryos, Dist FL Mes: *n*=45 nuclei from three embryos. (E) Graphic representation of tension sensor changes across the proximodistal limb bud axis, based on subsection D.

Spatial variations may also exist within structurally more comparable tissues. The limb bud is a compelling model system because distinct domains of patterning genes are expressed along its proximodistal axis (Cooper et al., 2011; Desanlis et al., 2020; McQueen and Towers, 2020), suggesting there may be a link between mechanisms that form the limb bud and pattern its differentiated tissues such as the skeleton. Evidence for mechanical heterogeneity within early limb bud mesoderm includes our earlier finding using magnetic tweezers that the proximal core is stiffer than the distal periphery (Zhu et al., 2020). Here, using light sheet microscopy to visualise CAG::H2B-miRFP703 (Gu et al., 2018), a far-red fluorescent nuclear reporter in live transgenic embryos, we found 3D nuclear sphericity is greatest in the proximal mesoderm of the E9.5 limb bud (Fig. 5B). At the resolution of unsegmented tissue, apparent tensions across NmpTS were also graded in a proximal-high to distal-low fashion (Fig. 5C, see Fig. 6 for quantification at cellular resolution), suggesting nuclear sphericity correlates with INM tension in this setting. To more directly examine the relationship between nuclear shape and nuclear sensor output, we evaluated nuclear shapes from the same confocal images we analysed by FLIM. NmpTS tension correlated with roundness whereas NespTS tension trended, insignificantly, with elongation (Fig. 5D). To distinguish fluorescence lifetimes along long and short axes of nuclei, we wrote a software supplement for FLIMvivo to segment nuclear envelopes in 2D (Fig. S8A). Consistent with previous NespTS findings *in vitro* (Arsenovic et al., 2016; Déjardin et al., 2020), neither NmpTS nor NespTS reported significant axis-dependent tension disparities, even under micro-aspiration (Fig. S8B-E), raising the possibility that the sensor proteins share the mechanical load around the nuclear envelope. A caveat to nuclear shape segmentation in 2D is the possibility of underestimating length/width ratios of elongated cells depending on the obliquity of optical sections. These data reveal similarities and differences in the responses of the two sensors and establish their ability to distinguish spatial variations of nuclear envelope tensions within a tissue.

To test for temporal changes in nuclear forces during development, we examined limb bud mesoderm at E9.5, E10.5 and E12.5. For NmpTS, proximal-high, distal-low patterns of measured lifetimes/forces persisted over time (Fig. 6A-D). Across those time points, mean NmpTS tensions diminished (Fig. 6B-D) while mean cross-sectional nuclear area enlarged (Fig. S9A,B), suggesting NmpTS tension correlates with smaller nuclei. For NespTS, proximodistal variation was not apparent until E12.5 when a proximal-low to distal-high, pattern was observed (Fig. 6B-D). That pattern is not unexpected given the different response of NespTS to cell shape changes that we identified by micro-aspiration. The spatial variations of the two sensors suggests there is a gradient of forces exerted across outer and inner layers of the nuclear envelope at E12.5 (Fig. 6E). Together, these findings suggest that nuclear mechanotransduction is a dynamic and nuanced process. We must contend with these nuances if we are to ‘decode’ the role of mechanical forces in gene expression and pattern formation.

## DISCUSSION

** **The tension sensor mouse strains we generated are complementary to an expanding suite of tools one can apply to examine mechanical mechanisms that drive cell behaviours and regulate gene expression *in vivo*. NmpTS and NespTS are sensitive and specific sensors capable of detecting exogenous and endogenous tensions across the nuclear envelope *in vivo*, but are not intended to test the specific characteristics of their host proteins. From one perspective, it is advantageous that the sensors are not functionally equivalent to their intact host proteins because the potential for phenotypes secondary to ubiquitous expression is avoided. By combining data from our reporter strains with analysis of cell and tissue properties and forces, chromatin conformation, and RNA and protein expression at single cell resolution, it is now possible to test mechanotransduction hypotheses in a quantitative fashion *in vivo*.

FRET controls do not necessarily behave as expected *in vivo*. In particular, headless/tailless sensor constructs do not uniformly report maximal FRET, minimal lifetime values, that would reflect a fully relaxed spring within the tension sensing module (this is also true *in vitro*; Arsenovic et al., 2016). Possibilities to explain those findings such as module position-dependent effects have been tested previously (Déjardin et al., 2020) and may also include unanticipated attachment of the headless/tailless protein end to other proteins or nearby membranes through lipophilic regions. By identifying the spatially independent output of the tailless/headless controls, we were able to distinguish the spatially dependent changes in the range of the full-length sensor constructs. The significantly lower variation of lifetimes generated by the tailless/headless controls validates the actuation of the sensors under endogenous forces. Although the availability of transgenic control strains was very useful to define the specificity of the sensors, their spatially stable behaviour implies that comparison to controls is not necessary for all future analyses, especially within a given tissue type.

While testing their responses to perturbations, we observed overlapping and distinct outputs between the two sensors. As expected, measurements using the esprin-2G and NEMP1 tension sensors confirmed that cytoskeletal forces are transmitted through the LINC complex and the nuclear lamina. Those observations are in keeping with our finding that NmpTS is under greater tension in relatively spherical nuclei since that trait corresponds to higher cortical tension. Interestingly, forced nuclear stretching resulted in distinct responses by the two sensors. Unlike the NmpTS that responded in a relatively linear manner, the NespTS exhibited a greater range of values. One possibility is that stress-induced cytoskeletal remodelling results in a relatively broad range of NespTS values.

The overall strategy we employed has pros and cons. Use of FRET-based sensors is advantageous since the output is largely not dependent upon expression levels. Major differences in expression are avoided by the strategy of knock-in to a ubiquitously expressed, single genomic site. However, the *in vivo* nature of our analyses presented particular challenges. Poor curve fitting resulting from the combination of low photon counts and background fluorescence (autofluorescence, background scatter) was a barrier to reliable FLIM analysis when we applied commercially available fitting software. Similar hurdles were encountered previously with an E-cadherin tension sensor in *Drosophila*, resulting in apparently poor sensitivity (Eder et al., 2017). Despite our tissues of interest being deep within mouse embryos, the software we developed through multiple rounds of iteration permits reliable and consistent FLIM analysis in living tissues and is applicable to a broad range of FLIM analyses.

The differences we observed between tissues such as the brain and heart as well as along the proximodistal limb bud axis correspond to previous measurements of tissue stiffness (Zhu et al., 2020). Those correlations imply that traction and contractile forces exerted by cells in stiffer environments are transmitted to the nuclear interior. Since gene expression domains co-vary with these properties along the proximodistal limb bud axis, we intend to examine the influence of forces upon transcription. Mechanical effects of force transmission to the nuclear interior is an exciting frontier in developmental and cell biology. Our ability to measure force transmission in animal systems opens doors to explore this area, and future technologies will no doubt improve the variety and precision of tools that measure how forces are transmitted to the nucleus.

## MATERIALS AND METHODS

### Transgenic mouse generation

All mouse breedings were performed in a barrier suite at Lab Animal Services at PGCRL and all procedures were approved by the Animal Care Committee in accordance with guidelines by the Canadian Council for Animal Care.

NmpTS/DO/TL were generated by PCR amplification of the tension sensor module from the Vinculin Tension Sensor (Grashoff et al, 2010) and ligation into *NEMP1*. NespTS/HL were purchased from Addgene (68127, 68128). Testing of constructs' proper subcellular localisation was done by Lipofectamine 2000 (Thermo Fisher Scientific, 11668030) transient transfection in 293T cells. EBFP and IRES were removed from the pR26-CAG/BFP-Dest plasmid (Addgene, 74282) and each construct was cloned into this ROSA26 targeting vector via gateway cloning (Thermo Fisher Scientific, 56484, 56481) through pDONOR (Thermo Fisher Scientific, 12536017) for mouseline generation. These constructs were sequenced to select reliable candidates for mouseline generation.

Chimeras were generated by traditional homologous recombination through aggregation and implantation of electroporated embryonic stem cells (ESCs) in wild-type embryos (The Centre for Phenogenomics, Toronto, Canada). Final ESCs were selected by PCR genotyping and copy number analysis (TCAG at the Peter Gilgan Centre for Research and Learning). Founders were confirmed by PCR genotyping and fluorescence imaging of crossings with various Cre lines. Constitutively active lines were generated by crossings with pCX-NLS-Cre (Mouse Genome Informatics, Tg(CAG-cre)1Nagy) or CMV-Cre (Jackson Laboratories, 006054) and littermates were compared visually and by weight. These lines were then inbred or outcrossed with CD1 to assess fertility.

### Microscopy

All imaging was performed in the Sickkids Research Institute Imaging Facility. Super resolution STED microscopy was performed on a Leica SP8 confocal microscope using 458 nm or 514 nm excitation wavelength with 592 nm or 660 nm depletion wavelength for sensor constructs *in vitro*. For immunofluorescence of embryonic tissue sections, 550 nm or 488 nm excitation wavelength with 592 nm or 660 nm depletion wavelength were used on a Leica SP8 Lightning Confocal. Huygens software was employed for deconvolution. Confocal images were captured on a Nikon A1R confocal microscope using Nikon NIS software for acquisition and Volocity for image processing. The FRET donor of the sensors is mTFP, so all FLIM data were acquired on a Nikon A1R confocal microscope connected to a PicoQuant pulse system with a 440 nm laser and 520/35 nm detector channel in Picoquant SymPhoTime 64 software. All FLIM images were acquired using transgenic mice in a wild-type background.

Live imaging was performed within 3 h of dissection on embryos in DMEM with 50% rat serum, at 37°C and 5% CO_2_ held in place with cheese cloth. In roller culture experiments, embryos were first placed in roller culture consisting of DMEM with 50% rat serum, 5% DMSO, and specified final drug concentrations and rolled for 1 h at 37°C in a 20% O_2_, 5% CO_2_ chamber. Aspiration experiments were performed on the A1R using a 90 µm diameter glass needle in a previously published device (Wen et al, 2017) which allows for precise modulation of pressure within the needle. The needle was moved along the surface of the coverslip to a position near the distal FL bud of live embryos for pre-aspiration imaging, then was moved to contact the bud and a precise pressure differential was applied for aspiration imaging (Fig. S7A).

In fixed sample imaging, α-aminB1 rabbit polyclonal antibody was used to label LaminB1 (1:1000, ab16048, Abcam) or α-O-GlcNAc mouse monoclonal antibody was used to label nuclear pores (1:500, NB300524SS, Thermo Fisher Scientific). In fixed *in vitro* samples, short (30 min., 4% PFA) fixation allowed for imaging of mTFP or Venus within sensor constructs, but longer fixation (o/n, 4% PFA) of *in vivo* samples required labelling of sensor constructs with mouse monoclonal α-GFP (1:500, sc-9996, Santa Cruz Biotechnology, Inc.).

### Sample sets and statistics

FLIM data were segmented into isolated nuclear membrane regions. Unless clarified otherwise in the figure legends, each dataset presented is nuclei from at least three separate embryos. With the exception of VinTFP which was fitted to entire fields of view, 15 nuclei, or as many clear nuclei as were available, per image were segmented for each region of each embryo. Further, segments with low quality data (as determined automatically by FLIMvivo) were rejected prior to statistical analysis.

Statistical analysis was done in Prism. With the exception of pregnancy rate comparisons, regression analyses, and variance comparisons, *P*-values were calculated using the *t*-test, applying Welch's correction to comparisons with significantly different standard deviations for datasets expected to be normally distributed and using the Mann–Whitney test for non-normally distributed datasets. For *t*-tests, four key assumptions (independence, normality, homogeneity of variance, and random sampling) were considered prior to test application. Pregnancy rate comparison *P*-values were calculated using the χ-square test. Variance comparisons were performed using *F*-tests. *P*-values greater than 0.05 were considered insignificant. In all experiments where tissues from the same embryos were compared, the values were normalised to the mean of all embryos and tissues for that experiment.

### FLIMvivo

FLIMvivo was written in Python and is available at https://www.github.com/HopyanLab/FLIMvivo. The basic FLIMvivo.py script takes a CSV data file and can perform either convolution or tail fitting applying either a mono-exponential or bi-exponential model. Segmentation masks for analysed FLIM images were generated using either the FLIMvivo or the FLIMfit (Warren et al., 2013) segmentation managers. The convolution option which uses fast Fourier transforms to accelerate convolution fitting and is the mode we use. One caveat here is that in order for the convolution scheme to behave properly, the data must be uniformly spaced in time, but this is not a problem in our situation.

A helper script, FLIMseg.py, is also included, which performs the sequence of fits described herein. It takes PicoQuant PTU files and TIF segmentation files, extracts decay data by segment (summing across multiple PTUs were they are supplied), then calls upon FLIMvivo to fit the extracted data. The script uses FLIMvivo first to fit the full field with a model of a Gaussian instrument response convoluted with a bi-exponential in order to establish an autofluorescence lifetime (Fig. 3C). It checks if the bi-exponential model seems appropriate and switches to a mono-exponential fit if necessary. Because the autofluorescence is likely unrelated to the sensors we wish to measure, this technique maximises the data available for a reliable fit. Finally, using either the autofluorescence lifetime from the full image field or a mono-exponential model, FLIMvivo is called again to fit for the signal lifetime in each segment (Fig. 3D). In this way, the autofluorescence and signal lifetimes are decoupled so that the signal fits are possible even when the per-segment data would not be good enough to fit for both simultaneously.

### Extended Methods

### FLIMvivo

We model the FLIM-FRET decay as a bi-exponential, with autofluorescent and signal components,

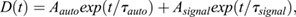
where the time constants are the means of the relevant distributions. So the microscope data we measure are the above decay function convoluted with an instrument response, which we model as a narrow Gaussian,

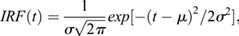
centred at μ with width σ. As an aside, a measured instrument response can be used here, but comparing the results using this technique with a measured response for a few data sets we see no significant difference in the fit signal lifetimes. We fit the full field to the convolution:

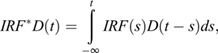
with the two amplitudes, two lifetimes, and two Gaussian parameters as fit parameters. Once complete, we can use the resulting autofluorescent lifetime and instrument response fitting any segment of the field with only the signal lifetime and two amplitudes as fit parameters. We additionally treat the end of the considered data range as a kind of fit parameter described below, so that we cut the data before background noise drives the fit away from the true signal value.

For our fitting algorithms we use a maximum likelihood fitting scheme assuming Poisson statistics, minimizing the ‘negative log-likelihood’ (NLL),

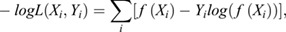
which is the proper statistical assumption for decay processes in contrast with the usual Gaussian assumption for chi-square fitting. In the case of high photon counts the two are both reasonable assumptions and give similar results, but as photon counts decrease the choice of statistical assumption becomes more relevant. As such, our choice is more justified for lower count data.

Finally, in fitting, we treat the end point (where the data is cut off) as fit variable, chosen by maximizing the ratio of best fit NLL to the square root of the number of data points. This way the data range is uniformly chosen by maximizing a measure of data contribution to our fit metric, thereby eliminating any potential bias introduced by choosing manually.

### Sensor PCR primers

All constructs were genotyped using these primers: Forward: CTCTGCTGCCTCCTGGCTTCT, WT Reverse: CGAGGCGGATCACAAGCAATA, Mutant Reverse: CCGCGAGCTGTGGAAAAAAAAGGG.

### Sensor sequences

#### NmpTS

ATGGCGGGAGGAATGAAAGTGGCGGTCTCGCCGGCAGTTGGTC CCGGGCCCTGGGGCTCGGGAGTCGGGGGC GGTGGGACAGTGC GGC TACTCTTGATCCTCTCCGGCTGCTTGGTCTACGGCACAGCT GAAACTGATGTAAATGTGGTCATGCTTCAGGAATCCCAA GTTTGTGAAAAGCGTGCCAGCCAACAATTCTGTTACACAAATGTGCTTATCCCAAAATGGCATGATATATGGACACGGATACAGATCCGAGTAAATAGTTCCAGATTGGTTCGAGTCACCCAGGTGGAGAATGAGGAGAAACTGAAGGAGCTAGAGCAGTTTAGTATCTGGAACTTTTTTTCCTCCTTTTTAAAAGAGAAATTGAATGACACCTATGTTAACGTGGGTCTATACAGCACAAAAACCTGCCTCAAAGTTGAGATTATAGAGAAGGACACCAAGTACAGTGTCATTGTGATCCGGAGATTTGATCCCAAACTCTTTCTTGTTTTCCTTCTTGGACTTATGCTATTTTTTTGTGGAGACTTGCTGAGCAGAAGTCAAATTTTCTACTACTCTACTGGGATGACTGTGGGAATTGTGGCCTCTCTGCTAATCATCATTTTTATACTATCTAAGTTTATGCCTAAGAAAAGTCCCATTTACGTCATCCTGGTGGGAGGCTGGTCTTTTTCTCTGTACCTCATTCAACTAGTTTTTAAAAATTTACAAGAGATCTGGAGGTGTTACTGGCAGTATCTTTTAAGTTATGTCCTCACAGTTGGATTCATGAGTTTTGCAGTATGTTACAAGTATGGGCCCTTGGAGAATGAACGAAGTATCAACCTGCTGACCTGGACCTTGCAGCTGATGGGCCTGTGTTTCATGTATTCTGGCATCCAGATACCACATATTGCCCTTGCCATTATCATCATTGCTCTTTGTACTAAGAACCTGGAACACGGATCCATGGTGAGCAAGGGCGAGGAGACCACAATGGGCGTAATCAAGCCCGACATGAAGATCAAGCTGAAGATGGAGGGCAACGTGAATGGCCACGCCTTCGTGATCGAGGGCGAGGGCGAGGGCAAGCCCTACGACGGCACCAACACCATCAACCTGGAGGTGAAGGAGGGAGCCCCCCTGCCCTTCTCCTACGACATTCTGACCACCGCGTTCGCCTACGGCAACAGGGCCTTCACCAAGTACCCCGACGACATCCCCAACTACTTCAAGCAGTCCTTCCCCGAGGGCTACTCTTGGGAGCGCACCATGACCTTCGAGGACAAGGGCATCGTGAAGGTGAAGTCCGACATCTCCATGGAGGAGGACTCCTTCATCTACGAGATACACCTCAAGGGCGAGAACTTCCCCCCCAACGGCCCCGTGATGCAGAAGAAGACCACCGGCTGGGACGCCTCCACCGAGAGGATGTACGTGCGCGACGGCGTGCTGAAGGGCGACGTCAAGCACAAGCTGCTGCTGGAGGGCGGCGGCCACCACCGCGTTGACTTCAAGACCATCTACAGGGCCAAGAAGGCGGTGAAGCTGCCCGACTATCACTTTGTGGACCACCGCATCGAGATCCTGAACCACGACAAGGACTACAACAAGGTGACCGTTTACGAGAGCGCCGTGGCCCGCAACTCCACCGACGGCATGGACGAGCTGTACAAGGGGCCAGGTGGTGCAGGGCCAGGTGGTGCAGGGCCAGGTGGTGCAGGGCCAGGTGGTGCAGGGCCCGGTGGTGCAGGTCCAGGTGGTGCAGGTCCAGGTGGTGCAGGTCCAGGTGGTGCTATGGTGAGCAAGGGCGAGGAGCTGTTCACCGGGGTGGTGCCCATCCTGGTCGAGCTGGACGGCGACGTAAACGGCCACAAGTTCAGCGTGTCCGGCGAGGGCGAGGGCGATGCCACCTACGGCAAGCTGACCCTGAAGCTGATCTGCACCACCGGCAAGCTGCCCGTGCCCTGGCCCACCCTCGTGACCACCCTGGGCTACGGCCTGCAGTGCTTCGCCCGCTACCCCGACCACATGAAGCAGCACGACTTCTTCAAGTCCGCCATGCCCGAAGGCTACGTCCAGGAGCGCACCATCTTCTTCAAGGACGACGGCAACTACAAGACCCGCGCCGAGGTGAAGTTCGAGGGCGACACCCTGGTGAACCGCATCGAGCTGAAGGGCATCGACTTCAAGGAGGACGGCAACATCCTGGGGCACAAGCTGGAGTACAACTACAACAGCCACAACGTCTATATCACCGCCGACAAGCAGAAGAACGGCATCAAGGCCAACTTCAAGATCCGCCACAACATCGAGGACGGCGGCGTGCAGCTCGCCGACCACTACCAGCAGAACACCCCCATCGGCGACGGCCCCGTGCTGCTGCCCGACAACCACTACCTGAGCTACCAGTCCAAGCTGAGCAAAGACCCCAACGAGAAGCGCGATCACATGGTCCTGCTGGAGTTCGTGACCGCCGCCGGGATCACTCTCGGCATGGACGAGCTGTACAAGGAATTCCCTATTCAGTGGCTGTACATCACCTGCAGAAAGGTGTGTAAGGGAGCAGAAAAGCCTGTTCCCCCTCGTCTCCTGACAGAAGAAGAATATCGGATACAAGGAGAGGTAGAAACCCGAAAGGCTTTAGAGGAGCTCCGAGAATTTTGTAACAGTCCAGACTGCTCTGCTTGGAAGACTGTTTCTCGAATCCAGTCTCCAAAAAGATTTGCTGACTTTGTGGAAGGCTCTTCCCACCTCACGCCAAATGAAGTTTCTGTCCATGAGCAGGAGTATGGATTAGGGAGCATTATTGCCCAGGATGAAATCTATGAGGAAGCATCCTCTGAGGAGGAGGACTCATATTCTCGGTGTCCTGCTATCACACAGAACAACTTTCTAACCTGA.

#### NmpTL

ATGGCGGGAGGAATGAAAGTGGCGGTCTCGCCGGCAGTTGGTCCCGGGCCCTGGGGCTCGGGAGTCGGGGGCGGTGGGACAGTGCGGCTACTCTTGATCCTCTCCGGCTGCTTGGTCTACGGCACAGCTGAAACTGATGTAAATGTGGTCATGCTTCAGGAATCCCAAGTTTGTGAAAAGCGTGCCAGCCAACAATTCTGTTACACAAATGTGCTTATCCCAAAATGGCATGATATATGGACACGGATACAGATCCGAGTAAATAGTTCCAGATTGGTTCGAGTCACCCAGGTGGAGAATGAGGAGAAACTGAAGGAGCTAGAGCAGTTTAGTATCTGGAACTTTTTTTCCTCCTTTTTAAAAGAGAAATTGAATGACACCTATGTTAACGTGGGTCTATACAGCACAAAAACCTGCCTCAAAGTTGAGATTATAGAGAAGGACACCAAGTACAGTGTCATTGTGATCCGGAGATTTGATCCCAAACTCTTTCTTGTTTTCCTTCTTGGACTTATGCTATTTTTTTGTGGAGACTTGCTGAGCAGAAGTCAAATTTTCTACTACTCTACTGGGATGACTGTGGGAATTGTGGCCTCTCTGCTAATCATCATTTTTATACTATCTAAGTTTATGCCTAAGAAAAGTCCCATTTACGTCATCCTGGTGGGAGGCTGGTCTTTTTCTCTGTACCTCATTCAACTAGTTTTTAAAAATTTACAAGAGATCTGGAGGTGTTACTGGCAGTATCTTTTAAGTTATGTCCTCACAGTTGGATTCATGAGTTTTGCAGTATGTTACAAGTATGGGCCCTTGGAGAATGAACGAAGTATCAACCTGCTGACCTGGACCTTGCAGCTGATGGGCCTGTGTTTCATGTATTCTGGCATCCAGATACCACATATTGCCCTTGCCATTATCATCATTGCTCTTTGTACTAAGAACCTGGAACACGGATCCATGGTGAGCAAGGGCGAGGAGACCACAATGGGCGTAATCAAGCCCGACATGAAGATCAAGCTGAAGATGGAGGGCAACGTGAATGGCCACGCCTTCGTGATCGAGGGCGAGGGCGAGGGCAAGCCCTACGACGGCACCAACACCATCAACCTGGAGGTGAAGGAGGGAGCCCCCCTGCCCTTCTCCTACGACATTCTGACCACCGCGTTCGCCTACGGCAACAGGGCCTTCACCAAGTACCCCGACGACATCCCCAACTACTTCAAGCAGTCCTTCCCCGAGGGCTACTCTTGGGAGCGCACCATGACCTTCGAGGACAAGGGCATCGTGAAGGTGAAGTCCGACATCTCCATGGAGGAGGACTCCTTCATCTACGAGATACACCTCAAGGGCGAGAACTTCCCCCCCAACGGCCCCGTGATGCAGAAGAAGACCACCGGCTGGGACGCCTCCACCGAGAGGATGTACGTGCGCGACGGCGTGCTGAAGGGCGACGTCAAGCACAAGCTGCTGCTGGAGGGCGGCGGCCACCACCGCGTTGACTTCAAGACCATCTACAGGGCCAAGAAGGCGGTGAAGCTGCCCGACTATCACTTTGTGGACCACCGCATCGAGATCCTGAACCACGACAAGGACTACAACAAGGTGACCGTTTACGAGAGCGCCGTGGCCCGCAACTCCACCGACGGCATGGACGAGCTGTACAAGGGGCCAGGTGGTGCAGGGCCAGGTGGTGCAGGGCCAGGTGGTGCAGGGCCAGGTGGTGCAGGGCCCGGTGGTGCAGGTCCAGGTGGTGCAGGTCCAGGTGGTGCAGGTCCAGGTGGTGCTATGGTGAGCAAGGGCGAGGAGCTGTTCACCGGGGTGGTGCCCATCCTGGTCGAGCTGGACGGCGACGTAAACGGCCACAAGTTCAGCGTGTCCGGCGAGGGCGAGGGCGATGCCACCTACGGCAAGCTGACCCTGAAGCTGATCTGCACCACCGGCAAGCTGCCCGTGCCCTGGCCCACCCTCGTGACCACCCTGGGCTACGGCCTGCAGTGCTTCGCCCGCTACCCCGACCACATGAAGCAGCACGACTTCTTCAAGTCCGCCATGCCCGAAGGCTACGTCCAGGAGCGCACCATCTTCTTCAAGGACGACGGCAACTACAAGACCCGCGCCGAGGTGAAGTTCGAGGGCGACACCCTGGTGAACCGCATCGAGCTGAAGGGCATCGACTTCAAGGAGGACGGCAACATCCTGGGGCACAAGCTGGAGTACAACTACAACAGCCACAACGTCTATATCACCGCCGACAAGCAGAAGAACGGCATCAAGGCCAACTTCAAGATCCGCCACAACATCGAGGACGGCGGCGTGCAGCTCGCCGACCACTACCAGCAGAACACCCCCATCGGCGACGGCCCCGTGCTGCTGCCCGACAACCACTACCTGAGCTACCAGTCCAAGCTGAGCAAAGACCCCAACGAGAAGCGCGATCACATGGTCCTGCTGGAGTTCGTGACCGCCGCCGGGATCACTCTCGGCATGGACGAGCTGTACAAGTGA.

#### NmpDO

ATGGCGGGAGGAATGAAAGTGGCGGTCTCGCCGGCAGTTGGTCCCGGGCCCTGGGGCTCGGGAGTCGGGGGCGGTGGGACAGTGCGGCTACTCTTGATCCTCTCCGGCTGCTTGGTCTACGGCACAGCTGAAACTGATGTAAATGTGGTCATGCTTCAGGAATCCCAAGTTTGTGAAAAGCGTGCCAGCCAACAATTCTGTTACACAAATGTGCTTATCCCAAAATGGCATGATATATGGACACGGATACAGATCCGAGTAAATAGTTCCAGATTGGTTCGAGTCACCCAGGTGGAGAATGAGGAGAAACTGAAGGAGCTAGAGCAGTTTAGTATCTGGAACTTTTTTTCCTCCTTTTTAAAAGAGAAATTGAATGACACCTATGTTAACGTGGGTCTATACAGCACAAAAACCTGCCTCAAAGTTGAGATTATAGAGAAGGACACCAAGTACAGTGTCATTGTGATCCGGAGATTTGATCCCAAACTCTTTCTTGTTTTCCTTCTTGGACTTATGCTATTTTTTTGTGGAGACTTGCTGAGCAGAAGTCAAATTTTCTACTACTCTACTGGGATGACTGTGGGAATTGTGGCCTCTCTGCTAATCATCATTTTTATACTATCTAAGTTTATGCCTAAGAAAAGTCCCATTTACGTCATCCTGGTGGGAGGCTGGTCTTTTTCTCTGTACCTCATTCAACTAGTTTTTAAAAATTTACAAGAGATCTGGAGGTGTTACTGGCAGTATCTTTTAAGTTATGTCCTCACAGTTGGATTCATGAGTTTTGCAGTATGTTACAAGTATGGGCCCTTGGAGAATGAACGAAGTATCAACCTGCTGACCTGGACCTTGCAGCTGATGGGCCTGTGTTTCATGTATTCTGGCATCCAGATACCACATATTGCCCTTGCCATTATCATCATTGCTCTTTGTACTAAGAACCTGGAACACGGATCCATGGTGAGCAAGGGCGAGGAGACCACAATGGGCGTAATCAAGCCCGACATGAAGATCAAGCTGAAGATGGAGGGCAACGTGAATGGCCACGCCTTCGTGATCGAGGGCGAGGGCGAGGGCAAGCCCTACGACGGCACCAACACCATCAACCTGGAGGTGAAGGAGGGAGCCCCCCTGCCCTTCTCCTACGACATTCTGACCACCGCGTTCGCCTACGGCAACAGGGCCTTCACCAAGTACCCCGACGACATCCCCAACTACTTCAAGCAGTCCTTCCCCGAGGGCTACTCTTGGGAGCGCACCATGACCTTCGAGGACAAGGGCATCGTGAAGGTGAAGTCCGACATCTCCATGGAGGAGGACTCCTTCATCTACGAGATACACCTCAAGGGCGAGAACTTCCCCCCCAACGGCCCCGTGATGCAGAAGAAGACCACCGGCTGGGACGCCTCCACCGAGAGGATGTACGTGCGCGACGGCGTGCTGAAGGGCGACGTCAAGCACAAGCTGCTGCTGGAGGGCGGCGGCCACCACCGCGTTGACTTCAAGACCATCTACAGGGCCAAGAAGGCGGTGAAGCTGCCCGACTATCACTTTGTGGACCACCGCATCGAGATCCTGAACCACGACAAGGACTACAACAAGGTGACCGTTTACGAGAGCGCCGTGGCCCGCAACTCCACCGACGGCATGGACGAGCTGTACAAGGAATTCCCTATTCAGTGGCTGTACATCACCTGCAGAAAGGTGTGTAAGGGAGCAGAAAAGCCTGTTCCCCCTCGTCTCCTGACAGAAGAAGAATATCGGATACAAGGAGAGGTAGAAACCCGAAAGGCTTTAGAGGAGCTCCGAGAATTTTGTAACAGTCCAGACTGCTCTGCTTGGAAGACTGTTTCTCGAATCCAGTCTCCAAAAAGATTTGCTGACTTTGTGGAAGGCTCTTCCCACCTCACGCCAAATGAAGTTTCTGTCCATGAGCAGGAGTATGGATTAGGGAGCATTATTGCCCAGGATGAAATCTATGAGGAAGCATCCTCTGAGGAGGAGGACTCATATTCTCGGTGTCCTGCTATCACACAGAACAACTTTCTAACCTGA.

## Supplementary Material

10.1242/biolopen.059656_sup1 Supplementary information
