# Supplementary material for: Transgenic force sensors and software to measure force transmission across the mammalian nuclear envelope in vivo
Source: Biol Open. 2022 Nov 9;11(11):bio059656. doi: 10.1242/bio.059656 (PMC9672859; doi:10.1242/bio.059656)
Supplement: Supplementary information [file biolopen-11-059656-s1.pdf]

Supplemental Figure 1

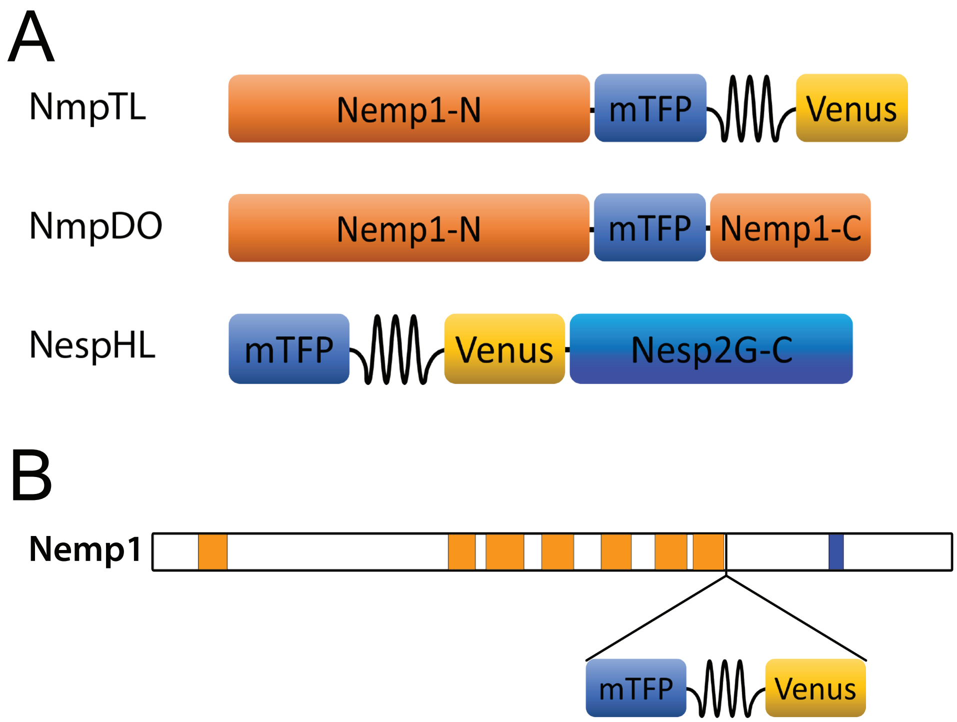

Fig. S1. Nuclear envelope tension sensor controls. (A) Nemp1 tailless (NmpTL) lacks the C-terminal, chromatin-interacting domain of Nemp1. Nemp1 donor only (NmpDO) lacks the flagelliform spring and the Venus fluorophore. NespHL is the previously developed Nesp2G headless control (30). (B) For NmpTS, the tension sensor module was inserted one amino acid C-terminal to the final transmembrane domain (orange domains). The blue domain represents the putative BAF binding site.

Biology Open • Supplementary information

Supplemental Figure 2

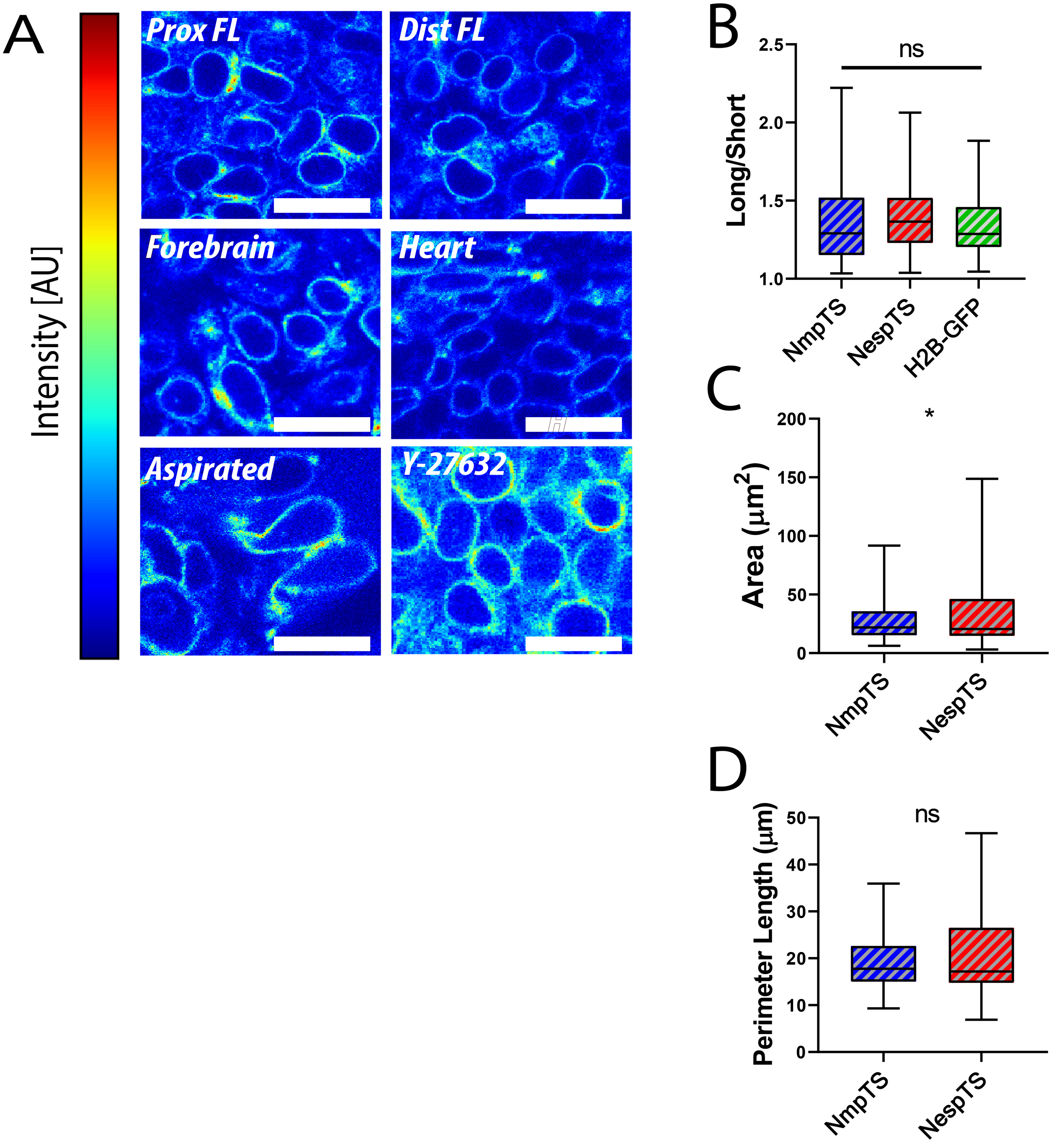

Biology Open • Supplementary information

Fig. S2. Experimental FLIM detection and transgenic nuclear size and shape data. A) Confocal FLIM images of NmpTS highlighted the nuclear envelope in various tissues and under various experimental conditions. The bottom two panels, Aspirated and Y-27632-treated, were captured from the distal FL. All images were obtained from living E9.5 embryos. Scale bars represent 25 µm. (B) E9.5 distal FL bud mesenchymal nuclei expressing NmpTS or NespTS shared similar shape profiles with H2B-GFP reporter. NmpTS vs. H2B-GFP: p=0.9659, Mann Whitney test; n=70 nuclei from 5 NmpTS embryos, n=45 nuclei from 3 H2B-GFP embryos. NespTS vs. H2B-GFP: p=0.0993, Mann Whitney test; n=105 nuclei from 7 NespTS embryos, n=45 nuclei from 3 H2B-GFP embryos. (C, D) Nuclear cross-sectional areas measured with NespTS at the ONM were slightly larger than those of nuclei labelled with NmpTS at the INM (E). The difference was insufficient to be detected by measuring nuclear perimeter lengths (F). Areas: p=0.0126, Welch's t-test; n=210 nuclei from 15 NmpTS embryos, n=283 nuclei from 19 NespTS embryos. Perimeters: p=0.1068, Welch's t-test; n=210 nuclei from 15 NmpTS embryos, n=283 nuclei from 19 NespTS embryos.

Supplemental Figure 3

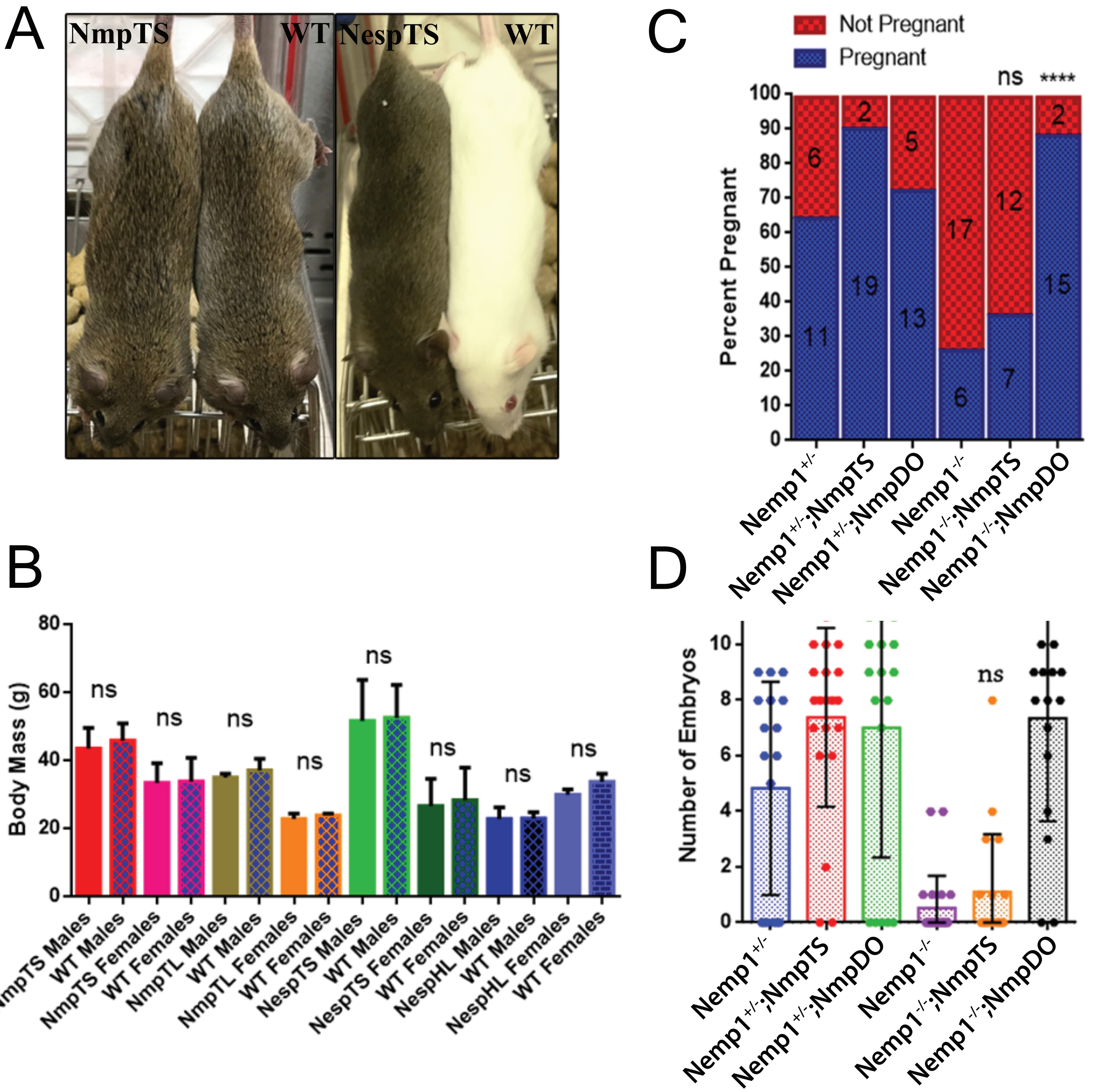

Fig. S3. Transgenic tension sensor mouse phenotypes. A) NmpTS and NespTS littermates exhibit-ed indistinguishable gross morphology. (B) Paired comparisons of sensor-expressing and non-expressing littermates revealed no weight differences at postnatal weeks 7-9. NmpTS: males: n=10 transgenics, 8 WT; females: n=9 transgenics, 8 WT; NmpTL: males: n=3 transgen-ics, 5 WT; females: n=8 transgenics, 10 WT; NespTS: males: n=4 transgenics, 6 WT; females: n=10 transgenics, 8 WT; NespHL: males: n=3 transgenics, 4 WT; females: n=3 transgenics, 4 WT. (C) Compared to Nemp1-/- females at E8.5-9.5, Nemp1-/-;NmpDO (****: $p \leq .0001$), but not Nemp1-/-;NmpDO ($p=.452995$), females were more frequently pregnant (as a binary mea-sure). n-values are given within the bars. (D) The NmpDO transgene rescued the diminished litter size of Nemp1-/- females at E8.5-9.5 (n as in C).

Biology Open • Supplementary information

Supplemental Figure 4

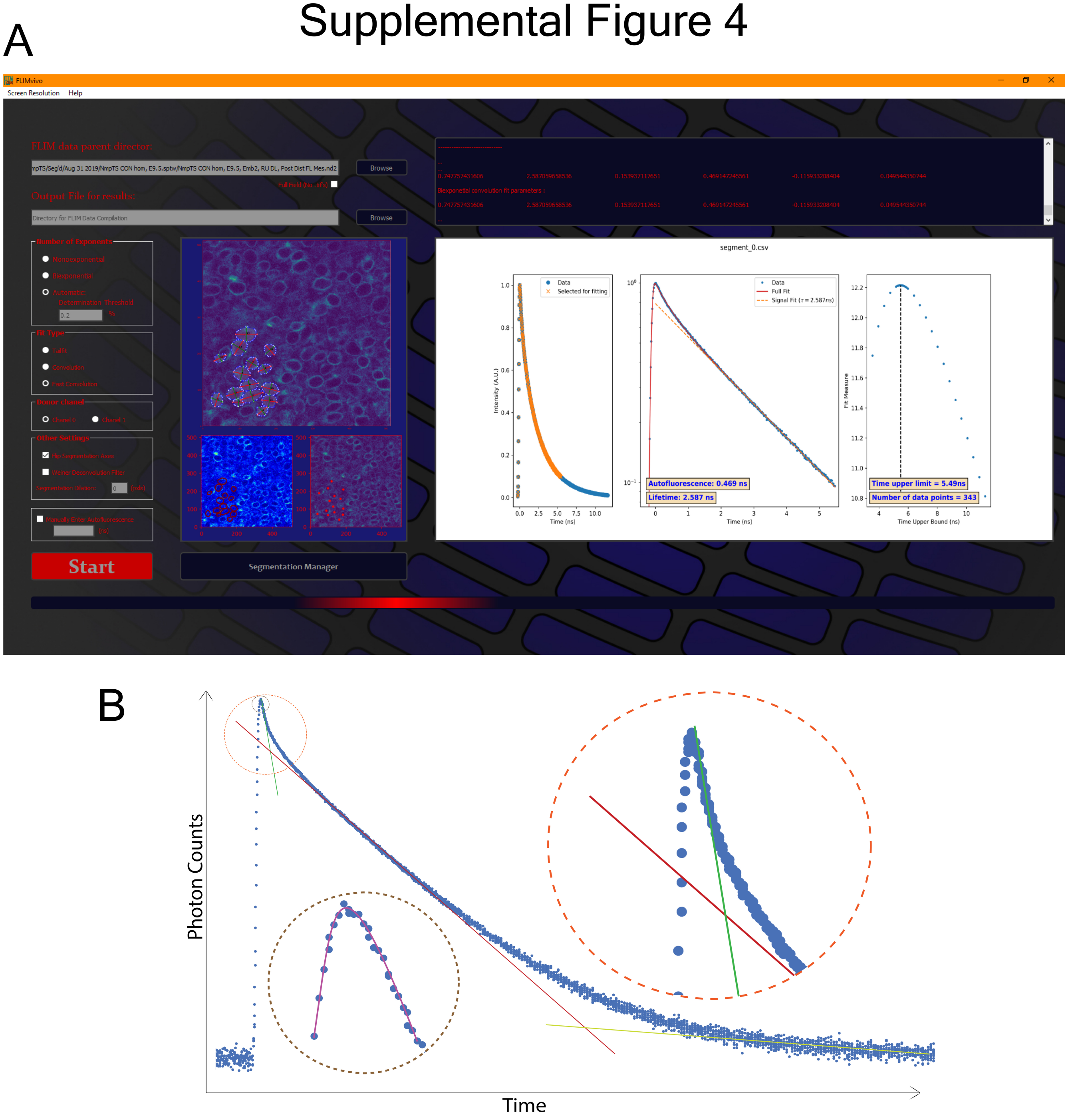

Biology Open • Supplementary information

Fig. S4. FLIMvivo user interface, segmentation and fitting. (A) We developed a user interface for FLIMvivo that allows for a broad array of FLIM experimental conditions (screenshot, top). Recommended settings are set to default. (B) FLIMvivo assesses the value of a bi-exponential convolution fit. It fits for the instru-ment response (purple line, brown dotted circle inset), autofluorescence (green line, orange dotted circle inset), and the sensor decay of the FRET donor (red line). Background light (yellow line) is removed by measuring goodness-of-fit across time points and removing data points beyond its peak. In samples with lower signal (donor)-to-noise (autofluorescence), a bi-exponential fit reflects the autofluorescence and donor decay curves (green, red lines). However, in samples with high signal-to-noise, a bi-exponential fit reflects the donor decay and background light curves (red, yellow lines), making a mono-exponential fit more appropriate combined with removal of the background tail (yellow).

Biology Open • Supplementary information

Supplemental Figure 5

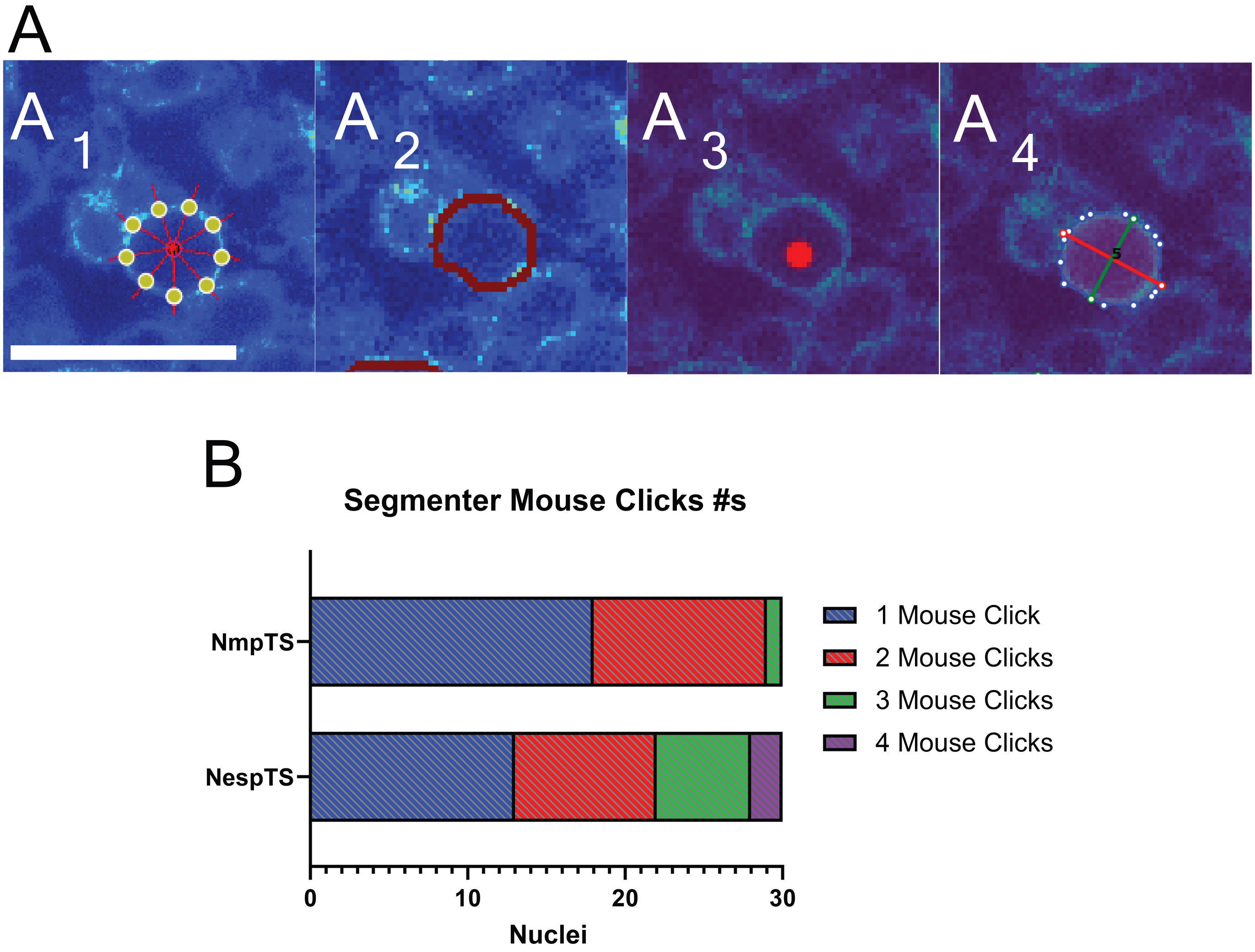

Fig. S5. FLIMvivo segmentation manager. (A) The user clicks inside a nucleus (A1); the segmentation manager detects the membrane by relative fluorescence intensity and saves the segmentation mask of pixel width chosen by the user (A2). Upon subsequent fitting with FLIMvivo, files are generated of the FLIM images overlaid with nuclear markers (A3) and length/width axes (A4). (B) 100% of nuclei segmented to test the recognition efficiency of the semi-automatic segmentation manager could be segmented with fewer than 5 mouse clicks. 85% were segmented with 2 or fewer mouse clicks. Differences in segmentation efficiency between NmpTS and NespTS were not significant (p=.0578, Mann Whitney test, n=30 nuclei from 3 embryos for each sensor) (C). All scale bars represent 25 µm.

Supplemental Figure 6

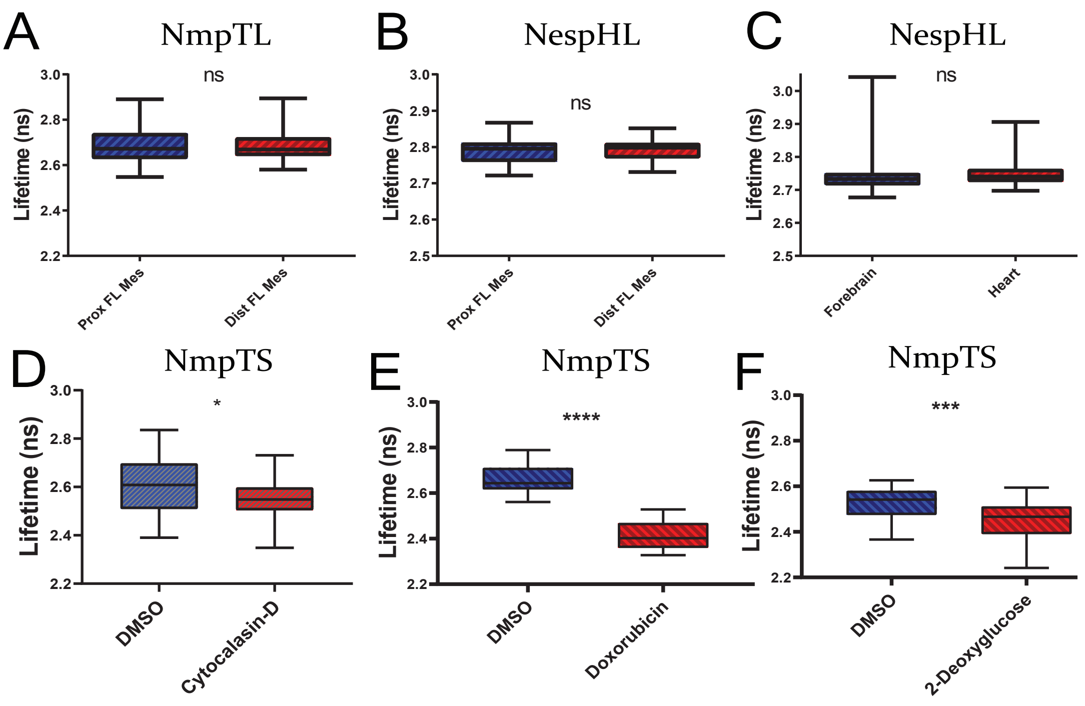

Fig. S6. Tailless and headless tension sensor, micro-aspiration, and chromatin drug controls. (A, B) Tailless and headless controls reported consistent and narrow lifetime ranges between proximal and distal aspects of the E9.5 limb bud. NespHL: Proximal Forelimb bud Mesoderm (Prox FL Mes): n=45 from 3 embryos; Distal (Dist) FL Mes: n=45 from 3 embryos; p=0.9565. NmpTL: Prox FL Mes: n=115 from 8 embryos; Dist FL Mes: n=110 from 8 embryos; p =.9699. (C) NespTS did not report significant differences between proximal and distal FL mesen-chyme, so NespHL donor fluorescent lifetime was compared in the forebrain and myocardium which were also insignificantly different (p=0.0996, t-test; Forebrain: n=75 from 5 embryos; Heart: n=75 from 5 embros). (D) Treatment of E9.5-10.5 NmpTS embryos with 1.25µg/mL Cytocalasin-D (an actin polymerisation inhibitor) significantly reduced donor lifetime (p=0.0193, t-test; n=30 from 2 embryos per condition). (E) Treatment of E9.5-10.5 embryos in roller culture with 0.5mM 2-Deoxyglucose and 0.1mM Sodium Azide to coagulate chromatin via ATP depletion significantly reduced the donor lifetime of NmpTS (p=0.0003, t-test; n=30 from 2 embryos for each condition). (F) Similarly, treatment with 10µM Doxorubicin to con-dense and fragment chromatin significantly reduced donor lifetime of NmpTS in E9.5 FL bud mesenchyme (p<.0001, t-test; n=15 from 1 embryo for each condition).

Biology Open • Supplementary information

Supplemental Figure 7

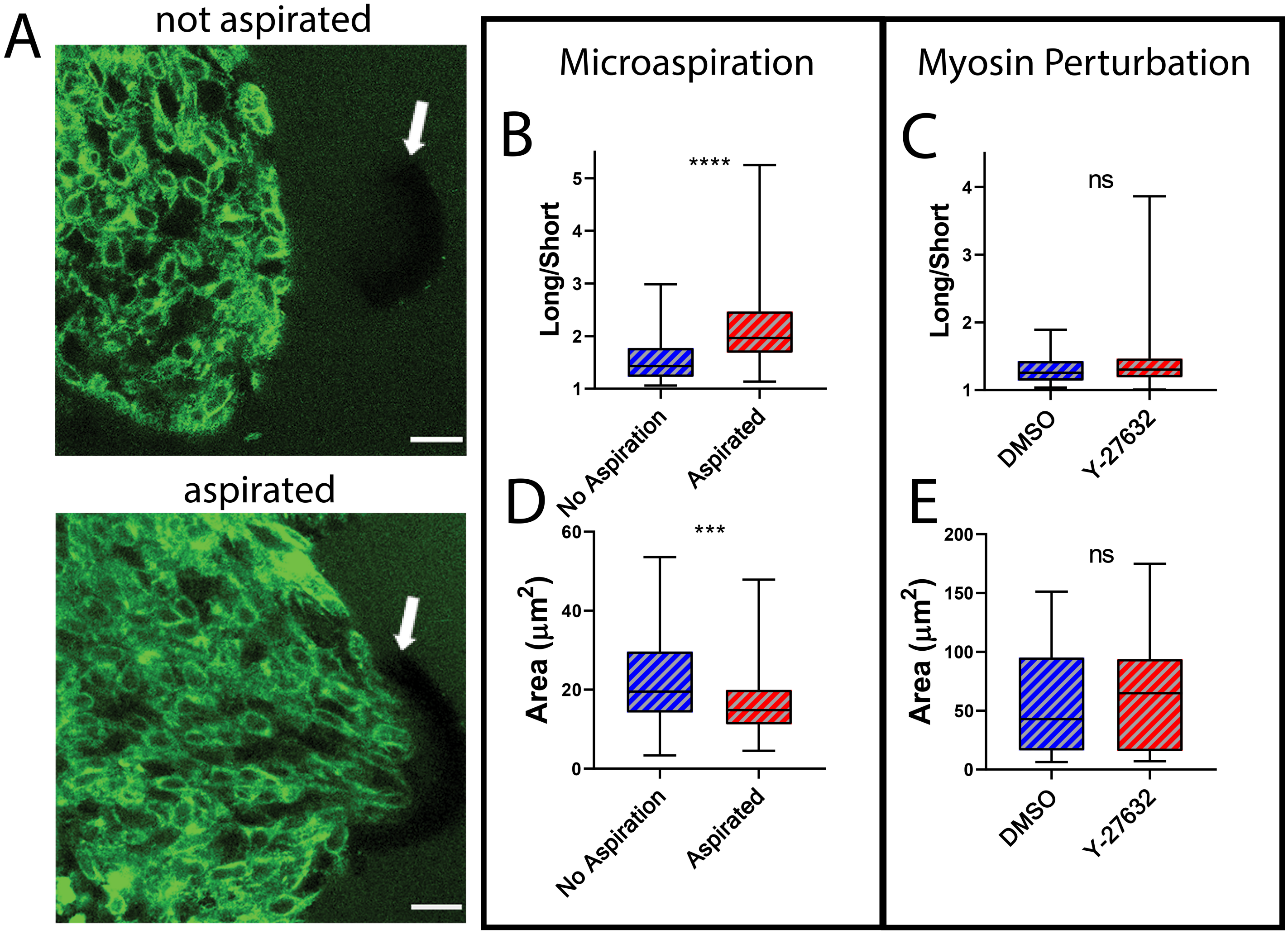

Biology Open • Supplementary information

Fig. S7. Nuclear deformation by micro-aspiration. (A) An example of tissue before and during micro-aspiration at 2500 Pa. Needle tip marked by white arrow. Scale bars rep-resent 25 μm. (B, C) Micro-aspiration by 2500 Pa, but not 5 μM Y-27632 treatment, elongated distal FL mesodermal nuclei marked with either sensor. For aspiration: $p<0.0001$, Mann Whitney test; n=125 nuclei from 9 unaspirated embryos, n=87 nuclei from 9 aspirated embryos. For Y-27632: $p=0.0837$, Mann Whitney test; n=90 nuclei from 6 DMSO-treated embryos, n=105 nuclei from 7 Y-27632-treated embryos. (D, E) Mi-croaspiration decreased cross-sectional areas of nuclei while Y-27632 had no significant effect. For microaspiraton: $p=0.0004$, t test; n=125 nuclei from 9 unaspirated embryos, n=87 nuclei from 9 aspirated embryos. For Y-27632: $p=0.5085$, t test; n=90 nuclei from 6 DMSO-treated embryos, n=105 nuclei from 7 Y-27632-treated embryos.

Supplemental Figure 8

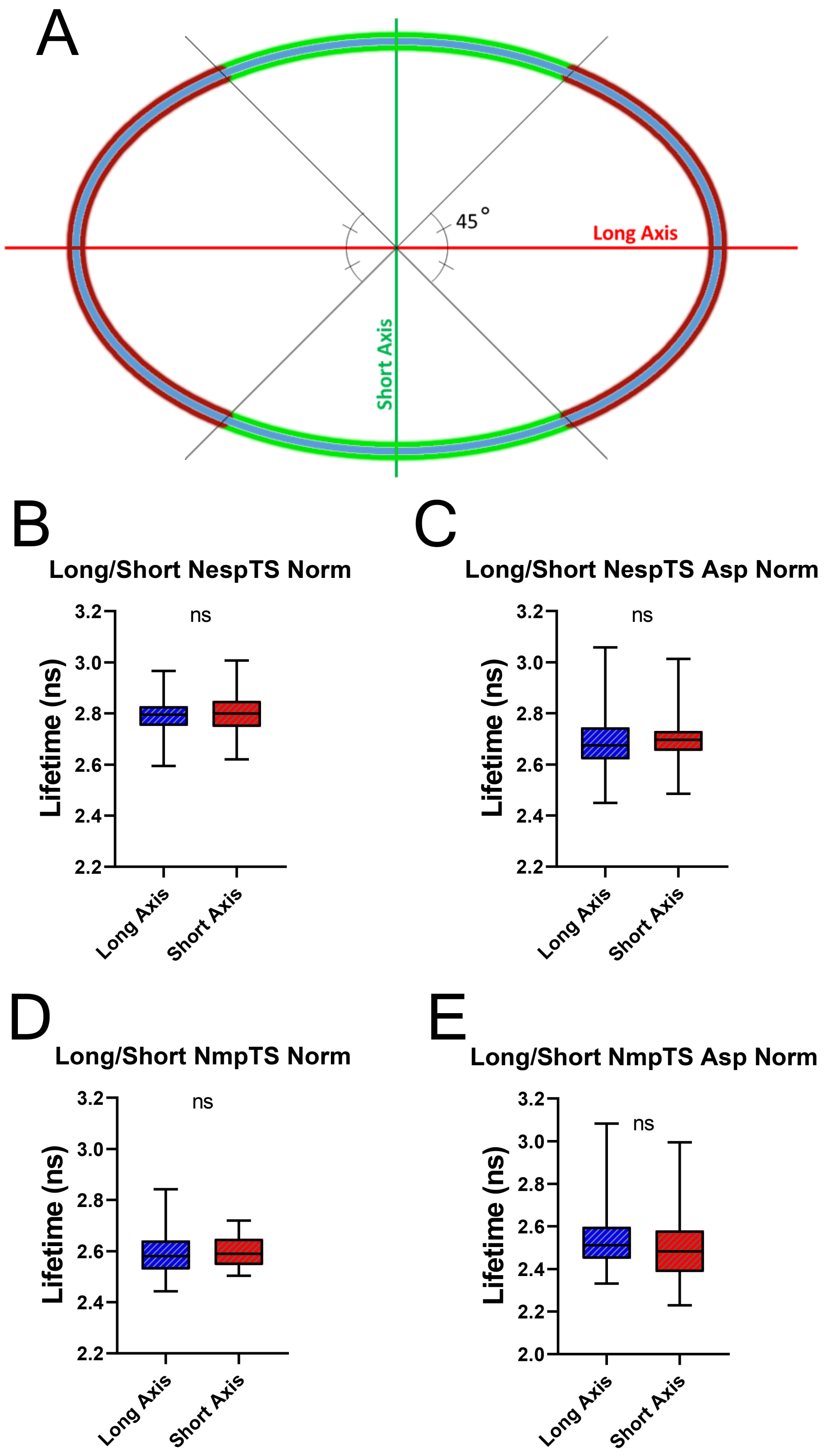

Fig. S8. Nuclear envelope segmentation suggests tension load sharing over nucle-ar periphery. (A) Illustration of nuclear membrane subdivision by modified FLIMvivo software package. The nuclear envelope is divided into ‘Long Axis’ and ‘Short Axis’ segments defined by 45˚ angles from the long axis of each nucleus which are then fit separately. (B-E) Neither sensor demonstrated a significant bias in tension between the long- or short-axes of the nuclear envelope under normal or micro-aspiration (as shown in Fig. 4) conditions. NespTS: $p=0.2715$, t-test, $n=105$ nuclear segments from 7 embryos per condition. NespTS Aspirated: $p=0.2721$, t-test, $n=79$ Long segments and $n=80$ Short segments from 8 embryos. NmpTS: $p=0.4932$, t-test, $n=33$ nuclear segments from 3 embryos per condition. NmpTS Aspirated: $p=0.3660$, t-test, $n=27$ Long segments and $n=33$ Short segments from 4 embryos.

Biology Open • Supplementary information

Supplemental Figure 9

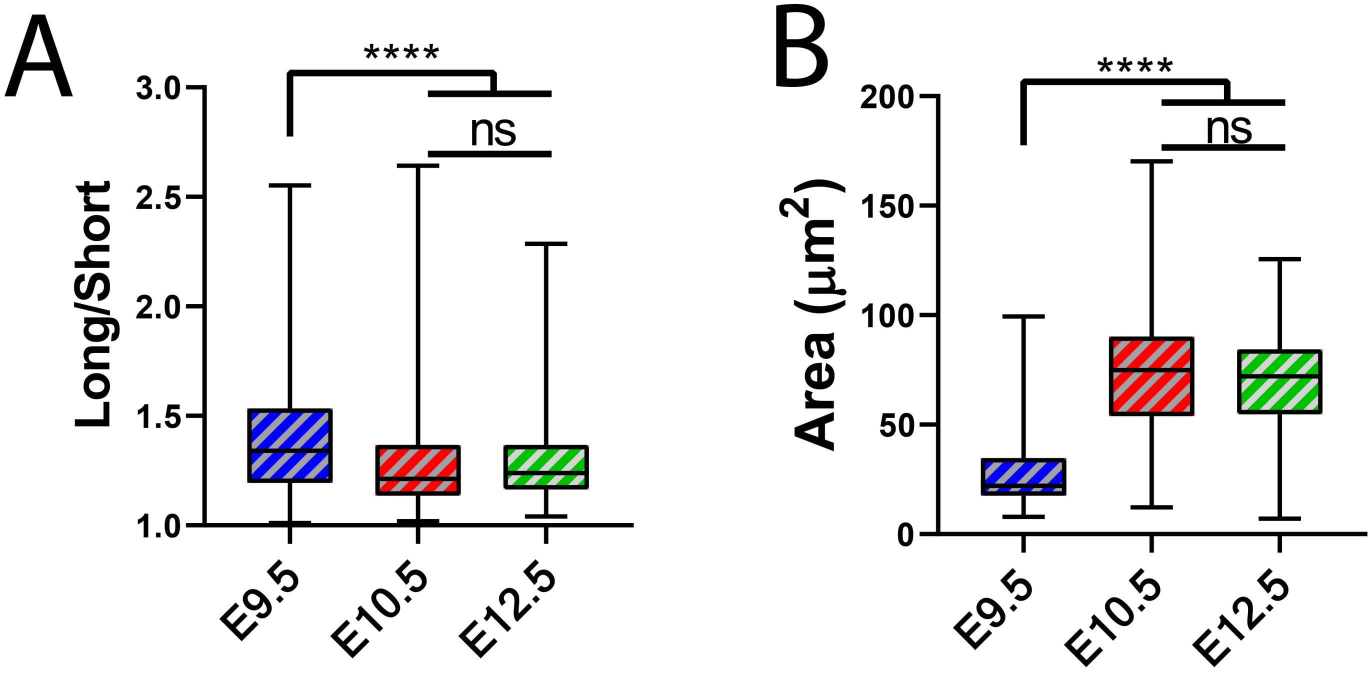

Fig. S9. Nuclear shape and area over time. In the forelimb bud between E9.5 and E12.5, mesenchymal nuclei acquire more rounded (A) and larger cross sectional area (B).
